# Novel Protein Kinase Signaling Systems Regulating Lifespan Identified by Small Molecule Library Screening Using *Drosophila*

**DOI:** 10.1371/journal.pone.0029782

**Published:** 2012-02-20

**Authors:** Stephen R. Spindler, Rui Li, Joseph M. Dhahbi, Amy Yamakawa, Frank Sauer

**Affiliations:** Department of Biochemistry, University of California Riverside, Riverside, California, United States of America; Brigham and Women's Hospital, Harvard Medical School, United States of America

## Abstract

Protein kinase signaling cascades control most aspects of cellular function. The ATP binding domains of signaling protein kinases are the targets of most available inhibitors. These domains are highly conserved from mammals to flies. Herein we describe screening of a library of small molecule inhibitors of protein kinases for their ability to increase *Drosophila* lifespan. We developed an assay system which allowed screening using the small amounts of materials normally present in commercial chemical libraries. The studies identified 17 inhibitors, the majority of which targeted tyrosine kinases associated with the epidermal growth factor receptor (EGFR), platelet-derived growth factor (PDGF)/vascular endothelial growth factor (VEGF) receptors, G-protein coupled receptor (GPCR), Janus kinase (JAK)/signal transducer and activator of transcription (STAT), the insulin and insulin-like growth factor (IGFI) receptors. Comparison of the protein kinase signaling effects of the inhibitors *in vitro* defined a consensus intracellular signaling profile which included decreased signaling by p38MAPK (p38), c-Jun N-terminal kinase (JNK) and protein kinase C (PKC). If confirmed, many of these kinases will be novel additions to the signaling cascades known to regulate metazoan longevity.

## Introduction

Protein kinase signaling cascades control most aspects of cellular function. As would be expected, many of the known mechanism for increasing the longevity of metazoans involve conserved protein kinase signaling cascades (reviewed in [1], [2]. In the best known example, reduction in insulin and/or insulin-like growth factor I signaling increases the lifespan of C. elegans, *Drosophila melanogaster*, and mus musculus (Reviewed in [1], [2]). Reduced signaling through these pathways may account for much of the life- and health-span effects of caloric restriction [1]–[3].

*Drosophila* is an attractive system for the identification and study of conserved pathways of lifespan regulation. Inhibitors of the mammalian receptor tyrosine kinases have been found to be effective against their insect orthologs [4]. Small molecule inhibitors typically bind the ATP binding pocket of signaling kinases, inhibiting their activity [5]. For example, the amino acid sequence of the human and *Drosophila* EGFR ATP binding fold are identical, and their surrounding amino acids are also highly conserved (Figure S1). Because of this similarity, the cancer therapeutics gefitinib and erlotinib are high affinity inhibitors of both the *Drosophila* and human EGFRs [4].

Herein we describe screening of a protein kinase inhibitor library (Table S1) for the effects of the inhibitors on *Drosophila* lifespan. We developed an assay which was able to utilize the small quantities of drugs typically available in chemical libraries. We found multiple kinase inhibitors, some targeting novel pathways, which were capable of extending *Drosophila* lifespan.

## Results and Discussion

### Protein kinase inhibitor library screening

**Figure 1:**
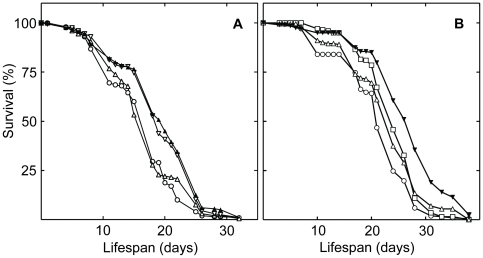
Rescreening results of drugs identified in an initial screening the Biomol protein kinase inhibitor library.

Chemical library screening using *Drosophila* posed a number of challenges. Chemical libraries are typically composed of small amounts of drugs. We developed methods for utilizing these small amounts to conduct lifespan assays with adult *Drosophila*. To increase the efficiency of the screenings, embryo densities [6], [7], culture temperature [8], [9], and dietary protein concentrations [8], [10] were adjusted to produce average lifespans of 20 to 30 days (Figure 1). These lifespans allowed rapid library screening. Our use of a single bottle throughout the assays might have shortened lifespans. However, the medium contained inhibitors of fungal and bacterial growth (propionic acid and Tegosept; Supporting Information S1) and the food-containing lids were changed twice-weekly. In no case did we find evidence of fungal or bacterial growth in the bottles or food. Never-the-less, we cannot exclude the possibility that the kinase inhibitors extended the lifespan of the flies by protecting them from the lifespan shortening effects of the screening conditions, rather than by extending lifespan *per se*. In future work, it will be important to monitor effects of the compounds under conditions where control flies exhibit longer lifespans.

Females have been used for longevity studies because of their greater response to protein and carbohydrate restriction [8]. However, males were used here to avoid the confounding effects of egg laying, which affects energy utilization, and therefore longevity.

The optimum concentration of an inhibitor in lifespan studies is related to its IC_50_, as well as its differential stability, uptake, metabolism, and excretion. Because these effects are difficult to predict, the inhibitors were tested at two concentrations, differing by 9-fold, termed *low* (0.5 mM) and a *high* (4.5 mM). Of the 80 inhibitors in the library (1), 19 showed a statistically significant increase in lifespan at one or both of these concentrations. Of these inhibitors, 17 passed one or more secondary screenings (Table 1). The secondary screenings also were used to determine the dose-response of the longevity effects. For example, 5 mM erbstatin analogue was found to increase lifespan by approximately 18% at 5 mM (Figure 1A), while 50 µM staurosporine increased lifespan by about 35% (Figure 1B).

**Table 1:** Results obtained with the inhibitors which passed the initial screening.

Biomol ID#	Compound name	Rescreening results^a^	Optimum concentration	Food consumption^b^
B1	PD-98059	27% (2)	300–1000 µM	NC
B3	SB203580	30.3% (3)	300 µM	NC
B6	Staurosporine	34.8% (3)	30–50 µM	NC
C4	Tyrphostin 1	19.0% (2)	100–300 µM	NC
C6	Tyrphostin AG1478	19.5% (2)	100 µM	NC
C7	Tyrphostin AG1295	22.5% (2)	300–500 µM	
C8	Tyrphostin 9	17.3% (3)	300–500 µM	NC
D1	AG-490	17.5% (2)	300 µM	
E1	HA-1004	18% (2)	1–10 µM	NC
E2	HA-1077	14.5% (2)	10 µM	NC
E5	KN-93	20% (3)	10–100 µM	
F4	PP2 AG1879	22.8% (1)	300 µM	
G1	Erbstatin analog	18% (1)	5 mM	NC
G2	Quercetin dihydrate	23% (3)	1 mM	NC
G8	SP600125	23.5% (2)	10 mM	NC
G9	Indirubin	8.2% (3)	3 mM	
H8^c^	Everolimus	16.6% (3)	3 mM	NC

a Percentages indicate the average percent increase in mean lifespan obtained in the number of independent trials indicated in parentheses. Because maximum lifespan (lifespan of the longest lived 10%) was variable due to the presence of occasional long-lived outliers, this metric was not used.

b NC indicates that the effects of the drugs on food consumption were not statistically different than control in FPAs and/or CAFE assays (see *Materials and Methods* for the details of the assays and statistical tests used). These FPAs used 6 control and 6 treated 8 ounce fly bottles containing 30 to 50 flies each. The absence of a notation indicates that the test was not performed for that drug. Where a range of optimum concentrations of the drug is given, the assay was performed at the highest concentration in the range.

c H8 was rapamycin (sirolimus) in the Biomol library. Because of its poor bioavailability, we tested everolimus, its more bioavailable derivative.

The targets of each of the 17 inhibitors, and their IC_50_s for these targets are shown in Table S2. The majority, at least 13 of these inhibitors, target signaling systems originating with one or more of the receptor tyrosine kinases (RTK; Tables S2 and S3). These kinases include the EGF, PDGF/VEGF, insulin/IGFI, JAK and GPC receptors. Downstream kinases which appear to be important for the longevity responses include p38, JNK and PKC (Tables 2 and S3).

**Table 2:** Summary of protein kinase activity studies.

Drug	Target(s)	Mek	ERK	p38	JNK	PKC	AMPK
AG-490 (D1)	JAK2		(NC)		a(↓)	NC	NC
Erbstatin Analogue (G1)	EGFR	NC	NC	↓	↑↑	↓	NC
HA-1004 (E1)	PKG	↓	NC	↑	↓	↓	NC
	PKA						
	CaMKII						
	PKC						
HA-1077 (E2)	ROCKI/II	↓	NC	NC	↓↓	NC	NC
	PRK2						
	MSK1						
Indirubin (G9)	GSK3β	s↑	NC	↓	NC	↓	NC
PD98059 (B1)	Mek	↓	NC	NC	↓	NC	NC
SB203508 (B3)	p38	↓↓	↓	s↓	NC	↓	↓
SP600125 (G8)	JNK	↑	NC	↓	↑↑	↓	NC
		(↓)	(↓)		(↓)		
Staurosporine (B6)	PKC	↓↓	↓	s↓	↓	↓↓	↓↓
		(↑)	(NC)		(↑)	(↓)	(↓)
Tyrphostin	EGFR	↓	NC	↑	↑	↓	NC
AG1478 (C6)		(↑)	(↑)		(↑↑)	(↓)	(↓↓)
Tyrphostin 9 (C8)	PDGFR	↑	↑↑	↓↓	↓	↓↓	NC
		(↓)	(↓)		(NC)		(↑↑)
Tyrphostin 1 (C4)	EGFR	↓	NC	↓	NC	↓	NC
		(↓)	(↓)		(NC)		(↑)
Consensus response in cells:		↓	NC	↓	↓	↓	NC
Down		7	2	7	5	10	2
Up		3	1	2	3	0	0
NC		1	8	2	3	3	10

a Down or up arrows indicate a decrease or increase in signal relative to control, respectively (Figure 2). NC indicates no change. A “s” adjacent to an arrow indicates that the change was small in magnitude. Arrows in parenthesis indicate changes found in Western blots of protein extracts from drug treated *Drosophila* (Figures 3 and 8).

### Lifespan extension by the inhibitors was not due to reduced caloric consumption

To investigate whether the effects on lifespan were the result of induced caloric restriction (CR), we determined the effects of 12 of the 17 inhibitors on food consumption using two of the best documented methods, *Fecal Plaque Assays* (FPAs; [11], [12]; Tables 2 and S2) and CAFE assays [13] (Table 2 and Table S5). The results obtained with the two assays are highly correlated (Pearson's coefficient = 0.9608; Figure S2). Drug treatment had no detectable effect on fecal plaque size (Figure S3 and Table S6; Ref. [14]). Both assays were utilized because the CAFE assay is a more widely used method of measuring food consumption, but the FPA more closely measured food intake under the conditions used for our lifespan studies. We found no effects on food consumption for any of the inhibitors tested using either assay (Summarized in Table 1; Tables S4 and S5). Together, these data indicate that the effects of the inhibitors on *Drosophila* lifespan do not involve CR.

### Effects of the inhibitors on intracellular protein kinase signaling

The changes in intracellular signaling induced by the inhibitors were investigated using protein extracts of control and drug treated S2 cells and *Drosophila*. Western blots were probed with antibodies specific for the phosphorylated and non-phosphorylated forms of the kinases. Changes in phosphorylation state were regarded as evidence of increased or decreased signaling by that kinase. Many signaling kinases were readily detected using extracts of cultured cells (Figures 2, 3, 4, 5). Representative Western blots are shown in Figures S4, S6, and S7. The position of these kinases in a consensus signaling network is illustrated in Figure 6. Fewer kinases could be detected in extracts of adult flies (Figures 7 and 8). Representative Western blots are shown in Figures S5 and S8. The phosphorylation states of some signaling kinases from adult flies were more difficult to detect than those from cultured cells, perhaps due to lower levels in the flies.

**Figure 2:**
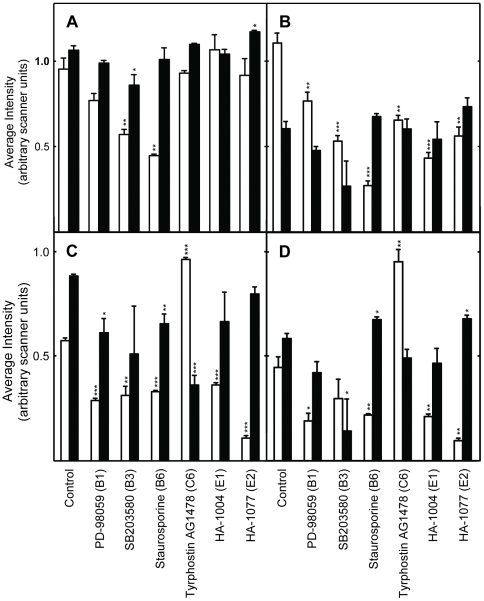
Effects of the protein kinase inhibitors on intracellular signaling in *Drosophila* S2 cells treated for 48 hours with the protein kinase inhibitors indicated at the bottom of the panels. Panel A shows data obtained using antibodies to phospho-Erk1/2 (white bars), and total Erk1/2 (black bars). Panel B shows data obtained using antibodies to phospho-Mek1/2 (white bars), and total Mek1/2 (black bars). Panel C shows data obtained using antibodies to phospho-p38 MAPK (white bars), and total p38 MAPK (black bars). Panel D shows data obtained using antibodies to phospho-JNK (white bars), and total JNK (black bars). The height of each bar represents the the signal normalized to the total amount of protein in each sample as judged by comparison to several apparently invariant protein bands observed on the blot by Ponceau S staining (representative staining is shown in Figure S4A). One asterisk indicates the changes were significant (P≤0.05); two asterisks indicates the results were highly significant (P≤0.01), and three indicates it is very highly significant (P≤0.001). See Figure S4A for representative Western blots and protein bands visualized using Ponceau S staining of membranes.

**Figure 3:**
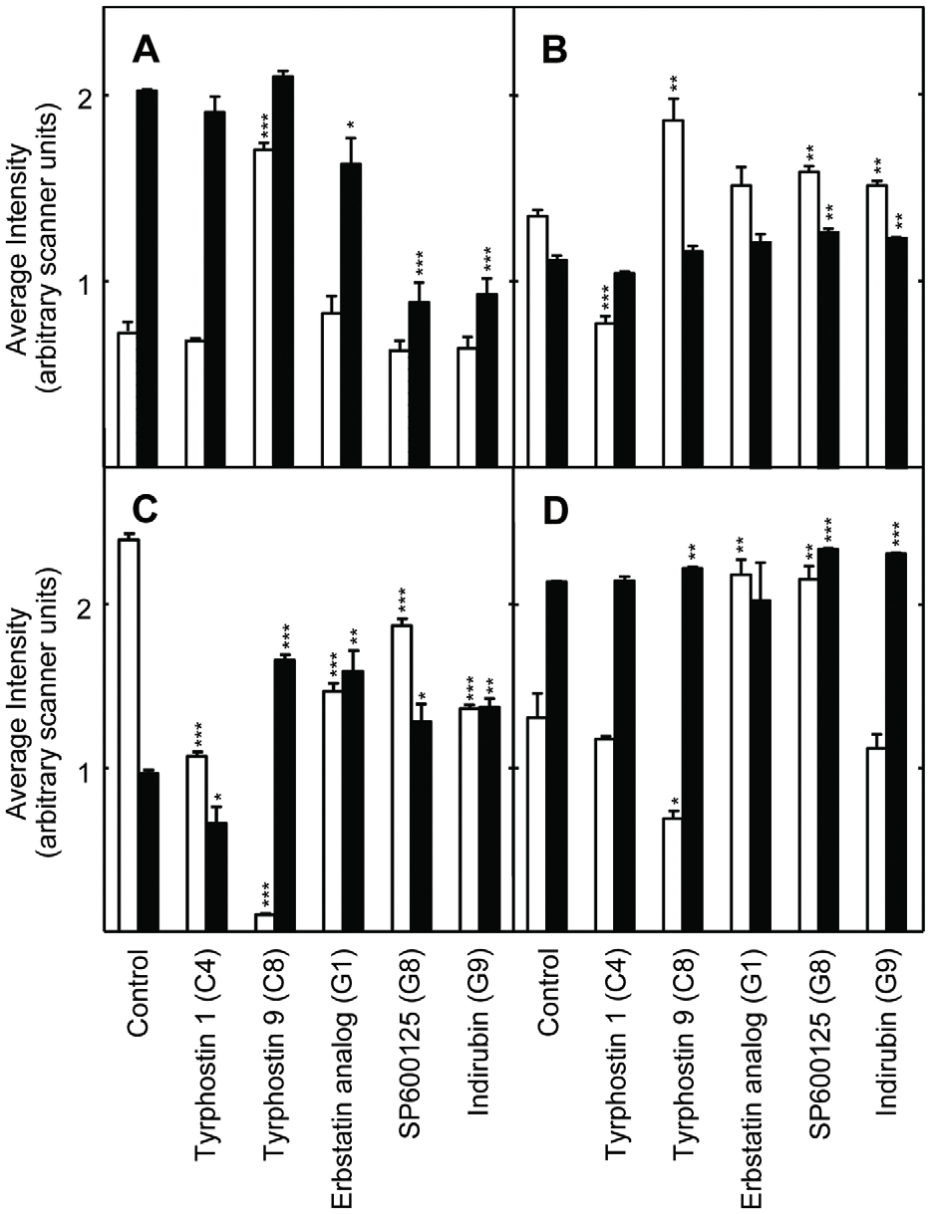
Effects of the protein kinase inhibitors on intracellular signaling in *Drosophila* S2 cells treated for 48 hours with the protein kinase inhibitors indicated at the bottom of the panels. The antibodies used to obtain data for each panel and the color coding of the data bars are as described for Figure 2. Data normalization was performed as described in Figure 2. The symbols indicating statistical significance are as in Figure 2. See Figure S4B and C for representative Western blots and Ponceau S stained protein.

**Figure 4:**
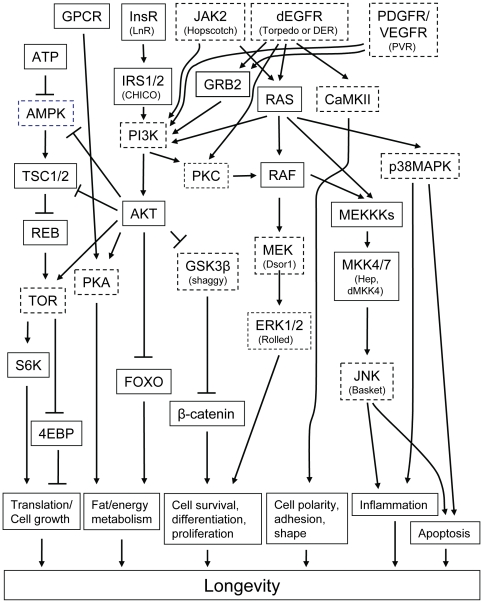
PKC activity determined using antibodies to phosphorylated substrates of PKC in extracts of S2 cells treated for 48 hours with the indicated protein kinase inhibitors. Panels A and B show results for different subsets of the inhibitors. The labeling and symbols are as described in the legend to Figure 2. See Figure S6 for representative Western blots.

**Figure 5:**
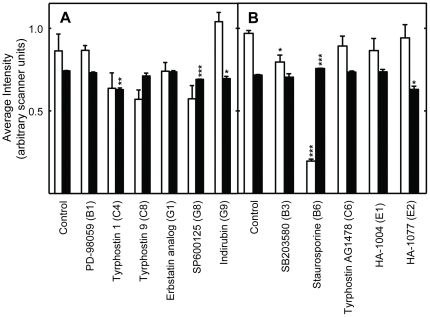
The level of phosphorylated AMPKα (white bars) and non-phosphorylated MPKα1+α2 (black bars) determined using Western blots prepared using protein extracts of S2 cells treated for 48 hours with the protein kinase inhibitors. Panels A and B show the data from one of two Western blots using control cells or cells treated with the indicated inhibitors. The labeling and symbols are as described in the legend to Figure 2. See Figure S7 for representative Western blots.

**Figure 6:**
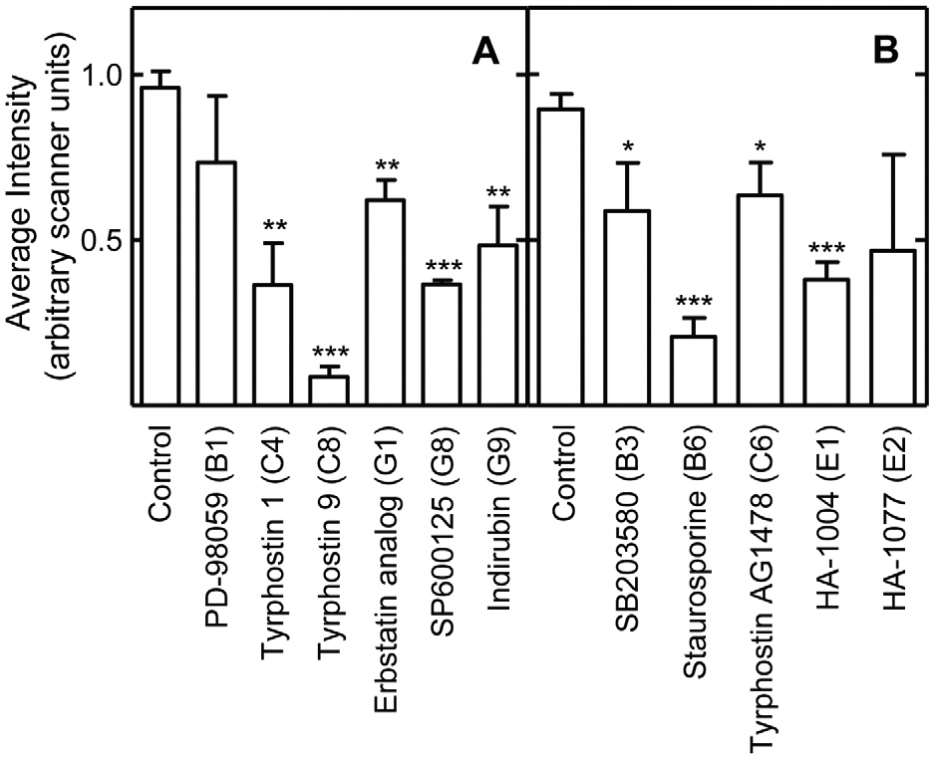
An abridged, consensus, protein-kinase signaling network assembled by examination of the *Drosophila* and mammalian literature.

**Figure 7:**
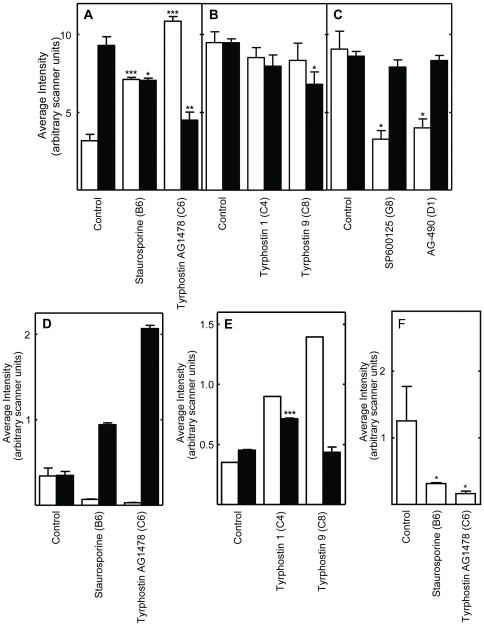
Effects of the indicated protein kinase inhibitors on site-specific phosphorylation determined using Western blots prepared using whole body protein extracts of *Drosophila* treated with various protein kinase inhibitors. Shown in panels A, B and C are the results obtained with antibodies specific to phosphorylated and total JNK; in panel D and E, the results obtained with antibodies to phosphorylated and total AMPK; and in panel F the results from antibodies to phosphorylated substrates of PKC. The inhibitors used in each study are indicated at the bottom of the panels. The symbols are as described in the legend to Figure 2. See Figure S5 for representative Western blots. In panel E, the control phosphorylated-AMPKα expression levels are derived from the averages of only 2 independent replicates, due to sample loss. All other data are derived from 3 independent replicates. See Figure S5 for representative Western blots.

**Figure 8:**
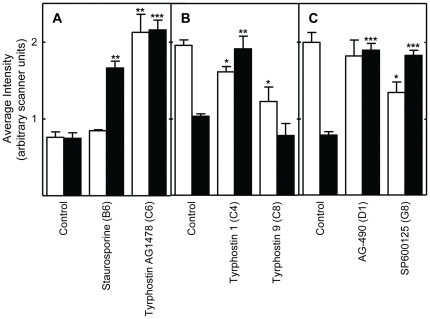
The effects of protein kinase inhibitors on the activation of ERK1/2 signaling in intact *Drosophila*. ERK1/2 activation in *Drosophila* fed the inhibitors in their food were determined using Western blots probed with a phosphorylation site- or total protein-specific antibody. The labeling and symbols are as described in the legend to Figure 2. Panels A through C represent data from 3 different Western blots. See Figure S8 for representative Western blots.

The effects of the inhibitors which extended lifespan on intracellular kinase signaling are summarized in Table 2. Considered together, the inhibitors reduced signaling by multiple RTKs, GPCR, JAK/STATs, and their downstream effectors. This inhibition produced changes in the activity of downstream kinases including Mek, p38, JNK, and PKC (Table 2). With few exceptions, signaling by ERK and AMPK were not strongly affected. In some cases, both the level of phosphorylation and the level of the kinase protein was altered by the inhibitors.

### EGFR

A novel result was that multiple inhibitors of EGFR tyrosine kinase activity extended *Drosophila* lifespan. As discussed above, the inhibitor binding sites of the human and *Drosophila* RTKs are structurally and functionally highly conserved, since most such inhibitors target the ATP binding fold (Figure S1; Refs. [4], [5]). We found that the EGFR inhibitors Tyrphostin AG1478 (AG1478), Erbstatin analogue, and Tyrphostin 1 produced similar, but not identical, effects in *Drosophila* S2 cells (Figures 2 and 3; Summarized in Table 2). All three drugs produced relatively modest effects on Mek1/2 and ERK1/2 signaling, which were either reduced or unchanged (Figures 2 and 3). These modest effects may have been due to the dosing regimen used. The highly selective Mek1/2 inhibitor PD-98059 significantly reduced Mek1/2 activity, and slightly, but not significantly, reduce the activity of its downstream target ERK1/2 (Figures 2 and 6; Data summarized in Table 2). Unexpectedly, inhibition of the PDGF receptor/VEGF receptor (PDGFR/VEGFR) homologue by tyrphostin 9 strongly activated Mek and ERK1/2 signaling, without changing their protein levels (Figure 3). These results suggest that PDGFR/VEGFR signaling represses EGFR activity in *Drosophila* cells in culture. However, *in vivo* tyrphostin 9 slightly reduced ERK1/2 signaling (Figure 8). These contrasting effects in cultured cells and flies are likely related to the differential uptake, metabolism or excretion of the drugs.

### JNK

The high affinity EGFR inhibitors AG1478 and Erbstatin analogue elevated JNK signaling in cells, without altering JNK protein levels (Figures 2 and 3). AG1478 also increased JNK signaling in flies (Figures 7A–C; Summarized in Table 2). Moderate JNK activation increases longevity and stress tolerance in Drosophila, C. elegans and mice ([15]–[18]; Reviewed in [19]). Thus, increased JNK activity may be the source of the lifespan effects of these inhibitors. In *Drosophila*, JNK activation reduces insulin (LnR in Drosophila) signaling by antagonizing IRS/Chico activation, leading to increased DAF-16/FOXO activity and increased lifespan [19]. As discussed in the paragraph above, the contrasting effects of SP600125 in flies and cells are likely related to its differential uptake, metabolism or excretion.

### PDGFR/VEGFR

Two PDGFR/VEGFR inhibitors, Tyrphostin AG1295 and Tyrphostin 9, extended *Drosophila* lifespan (Table 1). The PDGFR/VEGFR homologue in *Drosophila*, Pvr, signals through the canonical Ras/ERK pathway (Table S3). As discussed above, active Pvr appears to repress signaling through the *Drosophila* EGFR homologue (Torpedo/DER) in cell culture, since Tyrphostin 9 strongly upregulated both Mek and ERK signaling in cells (Figure 3). PDGFR inhibition by tyrphostin 9 also reduced signaling by p38, JNK, and PKC in S2 cells, but strongly induced AMPK signaling in flies (Figures 3, 6A and 7E). Increased AMPK signaling is closely associated with increased longevity in a number of metazoan systems [2]. Reduced p38 activity is not normally associated with increased longevity. However, in the studies reported here, lifespan extension is often accompanied by reduced p38 signaling (summarized in Table 2). For the reasons discussed in the *JNK* section above, reduced JNK activity is unlikely to contribute to the longevity effects of Tyrphostin 9. Thus, increased AMPK and p38 activity appear to be the likely reason for the positive effects of Pvr inhibition on lifespan.

### p38

p38 activity was downregulated in S2 cells by 7 of the 11 inhibitors which lengthened lifespan (Figures 2 and 3). SB203580 is a highly specific inhibitor of p38 (Table S2), and it both reduced the activity of its target and increased lifespan (Figure 2; Table 1). The p38 pathway has an evolutionarily conserved role in inflammatory signaling and the innate immune response in *Drosophila* [20]. Pro-inflammatory signaling mediated by p38 during aging has been associated with age-related pathology and attenuated lifespan [21]. In accord with these effects, 7 of the 11 inhibitors which extended lifespan reduced p38 signaling (Table 2). Thus, reduced p38 signaling is associated with increased longevity in our study.

### PKC

In general, inhibitors that increased lifespan decreased the level of PKC activity in S2 cells and intact flies (Figures 6 and 7F; Table 2). The decreased PKC signaling induced by AG1478, an EGFR inhibitor, in cells and flies (Fig. 5A and 6) is consistent with the reported activation of PKC by EGFR in mammalian cells, which suggests evolutionary conservation of this pathway between flies and mammals [22].

### AMPK

Two lifespan-lengthening inhibitors reduced AMPK activity *in vivo*, staurosporine and AG1478 (Figure 7D), and staurosporine also reduced AMPK levels in *Drosophila* cells *in vitro* (Figure 5). In contrast, two other inhibitors, tyrphostin 1 and tyrphostin 9, increased AMPK activity *in vivo* (Figure 7E). Increased, rather than reduced, AMPK signaling is normally associated with extended lifespan [23]. Enhanced AMPK signaling can extend the lifespan of both *Drosophila* and *C. elegans* [24], [25], while reduction of AMPK signaling can shorten *Drosophila* lifespan [26], [27]. Together, these results suggest that increased AMPK signaling contributes to the longevity effects of tyrphostin 1 and tyrphostin 9, but not to the effects of staurosporine and AG1478.

Genetic reduction of AMPK signaling was reported to induce hyperphagia in *Drosophila*, as determined using CAFE assays [26]. In contrast, we found no effects of staurosporine or AG1478 on food consumption (Tables 1 and S4). The reasons for these differences are not clear. It is possible that the kinase inhibitors did not reduce AMPK levels in the hypothalamic compartment which regulates feeding behavior.

### Staurosporine, HA-1004 and HA-1077

Staurosporine, a high affinity inhibitor of PKC (Table S2), inhibited signaling by PKC and AMPK *in vivo*, and signaling by all the other protein kinases tested *in vitro*, including JNK (Figures 2, 4, 5 and 7; Table 2). HA-1004 and HA-1077, which share many targets with staurosporine, also reduced signaling by JNK (Figures 2 and 4; Table 2). Reduced JNK signaling may be the mechanism for the lifespan extension by these inhibitors (as discussed above). However, it is important to note that staurosporine significantly increased JNK activity *in vivo* (Figure 7A–C). Thus, it is clear that other protein kinase targets must also be important for increased lifespan. For example, erbstatin analogue, indirubin, and tryphostin 1 did not decrease the JNK activity, but did increase lifespan. These drugs inhibited p38 activity, and this inhibition may explain their lifespan effects (as discussed above).

### Consensus response to the inhibitors

The consensus *in vitro* effects of the inhibitors on intracellular signaling are shown near the bottom of Table 2. No inhibitor which extended lifespan differed in more than two ways from this consensus response. The most highly conserved aspects of the consensus response are decreased PKC signaling, followed by decreased p38 and Mek signaling. These results suggest that these responses are key to increased *Drosophila* lifespan using the methods employed for these studies.

### Reasons for the differential effects of some kinase inhibitors in flies versus cell culture

Some inhibitors had different effects on protein kinase activity in cells and flies (Table 2). As discussed here, these differences most probably arise from differences in the uptake, bioavailability, stability, metabolism, or excretion *in vivo* versus in cell culture. Off target effects are also possible, but are less likely because of the high structural and functional conservation of the regions of the protein kinases targeted, as discussed.

### Effects of the kinase inhibitors in intact flies

Five of 6 of the inhibitors tested increased the relative concentration of ERK1/2 in intact *Drosophila*, although only one of these, the EGFR inhibitor AG1478, actually increased signaling by this protein (Figure 8). In one example of this effect, staurosporine doubled the amount of ERK1/2 protein present without changing the amount of phospho-ERK1/2. Two inhibitors actually increased total ERK1/2 but reduced the amount of active ERK1/2. Together, these results clearly illustrate that signaling activity can be regulated independently of the total amount of kinase protein present.

### Conclusions

Inhibition of EGFR, PDGF/VEGF receptor, GPCR, and JAK/STAT signaling can extend the lifespan of *Drosophila*. Comparison of the protein kinase signaling effects of the inhibitors *in vitro* defined a consensus intracellular signaling profile which included decreased signaling by p38, JNK and PKC. Many of these results are novel, and if confirmed, they will expand the number of signaling cascades known to regulate metazoan lifespan.

## Materials and Methods

### Protein kinase inhibitor library

A chemical library of 80 kinase inhibitors was purchased from Biomol International (now Enzo Life Sciences International, Inc., Plymouth Meeting, PA). Each inhibitor was supplied as 100 µl of a 10 mM solution in DMSO. This library was stored at −20°C until used. Wild-type Oregon-R-C *Drosophila* were obtained from the Bloomington *Drosophila* Stock Center (Department of Biology, Indiana University, Bloomington, IN, (http://flystocks.bio.indiana.edu/). All the protein kinases inhibitors used in the second round of testing (AG-490, erbstatin analog, HA-1004, HA-1077, hypericin, indirubin, KN-93, PD-98059, PP2 AG1879, SB203580, SP600125, staurosporine, tyrphostin 1, tyrphostin 9, tyrphostin AG1478) were from Enzo Life Sciences, except for tyrphostin AG1295, which was from Santa Cruz Biotech.

### Protein kinase inhibitor library screening for effects on *Drosophila* lifespan

In initial screening studies, 10 and 90 µl of each of the 10 mM protein kinase inhibitor stock solution obtained from Biomol was diluted to a final volume of 200 µl with DMSO, to make working solutions of 0.5 mM (low) and 4.5 mM (high). In initial studies, 10 µl aliquots of each solution were added to 0.5 ml of SY paste [28], mixed well with a spatula, and 0.25 or 0.125 ml of the mixture spread evenly onto the surface of 35×10 mm Petri dish lids (Falcon) containing a 5.5 ml plug of 10 g/L agar and 10 mM Tegosept. Controls utilized 10 µl of DMSO mixed and spread as described above. The filled Petri dish lids were used as bottle closures by placing them over the opening of 8 ounce, plastic fly bottles (Genesee Scientific), and securing them with tape. For the reasons enumerated in *Results and Discussion*, later screening studies were performed using Petri-dish bottle closures prepared by carefully applying 10 µl of DMSO (control), the low or the high concentration protein kinase inhibitor working solutions directly to the surface of the SCM-agar (see Supporting Information S1) containing Petri-dish bottle closures, and spreading the solutions carefully over the entire surface of the SCM-agar with the edge of the micropipette tip. In a later variation, the drug aliquots were mixed with 0.5 ml of SCM before they were layered onto the SCM agar-containing bottle closures. The SCM-agar closures were prepared beforehand, and stored at 4°C for up to several weeks before being warmed to room temperature for use. Thirty to 50 flies in each of 4 to 8 bottles (200 to 240 flies total) were used for each control or treatment group. The bottles were closed with Petri dish lids secured with tape. The walls of the fly bottles were pierced with small air holes, and the bottles incubated at 60% humidity, on a 12∶12 hour light∶dark cycle, with the lid closures on top to minimize evaporative drying. As discussed in *Results and Discussion*, a few initial longevity studies were conducted at 29°C. The temperature was reduced to 25°C, and this was used for most of the studies. The Petri-dish bottle closures were replaced twice weekly. The number of flies living at each time point was determined by visual inspection. CO_2_ anesthesia was not used after the initial sorting of the males. Studies using dyes in the 10 µl DMSO overlays indicate that the drug diffuses approximately 1–2 mm into the SCM-agar in 72 hours at 25°C. See Supporting Information S1 for more detail. Lifespan differences between vehicle and drug treated groups were determined using survival analysis followed by the Logrank test (SAS).

### Quantification of fly food intake

Food intake was quantified in two ways. Capillary feeder (CAFE) assays [13] were modified for 30 flies in eight ounce plastic fly bottles with four, 100 µl graduated glass microcapillary pipettes containing 5% sucrose, 5% autolyzed yeast extract (Fisher), and 0.01% (v/v) DMSO either alone (control) or containing the concentration of drug indicated in the text. A small amount of red food coloring was added to facilitate measurements. The cotton Flug bottle closures were saturated with 25 ml of reverse osmosis purified water to maintain humidity during the 24 hour incubation at 25°C.

FPAs were performed essentially as described [12], with minor modifications. Briefly, standard SCM-agar Petri dish lids were prepared. One-half ml of SCM without agar was mixed with 10 µl of DMSO without (control) or with 10 µl of the indicated concentration of drug and one drop of red food coloring, and evenly spread over the surface of an SCM-agar lid. The lids were used to feed bottles of 50 flies for 24 hours. After removal of the lids and the flies, 5, 4×4 cm squares were randomly marked on the sides of each bottle near the midline, and the numbers of red plaques counted. Fly bottles from FPAs were positioned under a Celestron Handheld Digital Microscope, Model #44302-A, and their diameter determined using the software provided (n = 40 for each condition). The mean number of plaques per square centimeter and plaque diameter was compared by t-test or one way ANOVA using GraphPad Prism.

### Protein kinase inhibitor treatment of S2 cells

Drosophila S2 cells (a gift from DRSC at Harvard Medical school) were grown at 28°C in 1× Schneider's Drosophila medium (Gibco) supplemented with 10% FBS. Aliquots of 2×10^6^ cells were seeded in 75 cm^2^ plastic tissue culture bottles and grown for 1 day before the kinase inhibitors, dissolved in DMSO, were added. A second inhibitor dose was added on the third day. DMSO treated cells were used as control. Cells were collected on the fourth day, snap frozen in liquid nitrogen, and stored at −80°C. The final dose of each inhibitor was: Tyrphostin AG1478, 40 µM; tyrphostin 1, 20 µM; tyrphostin 9, 20 µM; PD-98059, 40 µM; staurosporine, 0.04 µM; SB- 203580, 4 µM; HA-1004, 80 µM; HA-1077, 20 µM; erbstatin analog, 20 µM; SP600125, 20 µM; Indirubin, 20 µM.

### Western blotting of proteins isolated from *Drosophila*

Controls or *Drosophila* treated with protein kinase inhibitors at their optimum dosage for lifespan extension were snap frozen, and stored at −80°C. Four bottles per treatment group, with 50 male flies per bottle, were powdered under liquid nitrogen with a mortar and pestle. Soluble and membrane bound proteins were fractionated using a ProteoJET Membrane Protein Extraction Kit (www.fermentas.com) as described by the supplier, except that the Cell Permeabilization and Membrane Protein Extraction Buffers contained protease inhibitor cocktail (10 µl per ml; Sigma #P8340). After extraction, the total soluble and membrane bound fractions were combined and used for Western blot analysis. Separate extraction of soluble and membrane bound fractions gave a higher protein yield and intact proteins relative to any total protein extraction method used. Proteins (100 µg per lane) were separated by SDS-PAGE, transferred to a PVDF membrane, and images developed by ECL chemiluminescence according to standard procedures. The membranes were probed using antibodies directed against phospho-p38 MAPK (Thr180/Tyr182) (Cell Signaling, #9216), p38 (Santa Cruz Biotechnology, #sc-15714), phospho-p44/42 MAPK (ERK1/2; Thr202/Tyr204) (Cell Signaling, #9101), p44/42 MAPK (Erk1/2) (Cell Signaling, #4348), phospho-Mek1/2 (Ser 218/Ser222) (Santa Cruz Biotechnology, #sc-7995), MEK1/2 (Cell Signaling, #9122), phospho-SAPK/JNK (Thr183/Tyr185) (Cell Signaling, #4668), JNK (Santa Cruz Biotechnology, #sc-571), phospho-AMPKα (Thr172) (Cell Signaling, #4188), AMPKα_1_+AMPKα_2_ (Abcam, #ab80039), phospho-(Ser) PKC Substrate (Cell Signaling, #2261), calnexin (Abcam, #ab75801), and β-actin (Abcam, #ab20272). Secondary antibodies conjugated to horseradish peroxidase, were either goat anti-rabbit IgG (Abcam, ab6721# ), rabbit anti-mouse IgG (Abcam, ab6728#), or donkey anti-goat IgG (Santa Cruz Biotechnology, sc-2020#). Protein loading was determined using Ponceau S staining of PVDF membranes and unregulated protein bands were identified by visual inspection of the stained membranes. Ponceau S staining was utilized rather than antibody probing for a single “control protein” because such probing would limit our observations to this single control. Differential regulation of the control by one of the many treatments could be mistaken for a variations in loading. In contrast, observation of a large number of proteins on the stained membrane reduces the likelihood of this possible artifact. Band intensity was quantified with NIH ImageJ or Kodak Molecular Imaging Software, Standard Edition (Carestream Health, Inc.). Phosphorylation levels were normalized for protein loading and the data plotted using GraphPad Prism (version 5.01, www.graphpad.com). Statistical significance between DMSO treated and kinase inhibitor treated samples was determined with a two-sample t-test, assuming equal variances. In multiple studies using a variety of protein isolation techniques, only the signals for the antibodies utilized in Figures 7 and 8 (phospho-(Ser) PKC substrate, phospho-JNK, phospho-ERK1/2, and β-actin) reproducibly produced signals above background using proteins isolated from intact flies.

### Western blotting of protein kinase inhibitor treated S2 cells

Protein extracts were prepared by sonication of cell pellets in 350 µl of SDS buffer (50 mM Tris-HCl, pH 6.8, 2% SDS, and 10% glycerol) containing 5 µl/ml protease inhibitor cocktail (Sigma #P8340) and 5 µL/ml phosphatase inhibitor cocktails 2 and 3 (Sigma #P5726 and P0044) for 20 sec at a setting of 7 (Sonifier Analog Cell Disruptor, Model S-450A), and centrifugation at 16,000× g for 20 min. Supernatants were collected and protein concentrations determined using a Pierce BCA Protein Assay Kit (Thermo Scientific) as described by the manufacturer. Proteins (100 µg per lane) were separated by SDS-PAGE, transferred to a PVDF membrane, membranes probed and the data processed as described above for proteins isolated from *Drosophila*.

## Supporting Information

Figure S1 Sequence alignment of the EGFR proteins from human and Drosophila melanogaster.
(TIF)

Figure S2 There is a strong correlation between food consumption measured by the CAFE and FPAs.
(TIF)

Figure S3 Representative plaques from control and drug treated *Drosophila*.
(TIF)

Figure S4 Representative Western blot results of the effects of protein kinase inhibitors on intracellular signaling in *Drosophila* S2 cells.
(TIF)

Figure S5 Representative Western blot results of the effects of the protein kinase inhibitors indicated at the top each figure on intracellular signaling in intact *Drosophila*.
(TIF)

Figure S6 Representative PKC-substrate serine phosphorylation measured using an antibody specific for phospho-(Ser) PKC substrates.
(TIF)

Figure S7 The results of representative Western blots probed with antibodies directed against the phosphorylated and non-phosphorylated forms of AMPKα.
(TIF)

Figure S8 The results of representative Western blots probed with antibodies directed against the phosphorylated or non-phosphorylated forms of ERK1/2 or β-actin.
(TIF)

Table S1 Comprehensive list of the compounds present in the BioMol kinase inhibitor library.
(DOC)

Table S2 Reported targets of the protein kinase inhibitors confirmed to extend Drosophila lifespan.
(DOC)

Table S3 Summary of the pathways regulating Drosophila lifespan identified through these studies.
(DOC)

Table S4 Summary of food consumption of treated and untreated flies as measured by FPAs.
(DOC)

Table S5 Summary of food consumption of treated and untreated flies as measured using CAFE assays.
(DOC)

Table S6 Summary of the drug effects on fecal plaque size.
(DOC)

Supporting Information S1 Supporting Materials and Methods.
(DOC)
