# Supplementary figures and images for: Novel Protein Kinase Signaling Systems Regulating Lifespan Identified by Small Molecule Library Screening Using Drosophila
Source: PLoS One. 2012 Feb 20;7(2):e29782. doi: 10.1371/journal.pone.0029782 (PMC3282711; doi:10.1371/journal.pone.0029782)

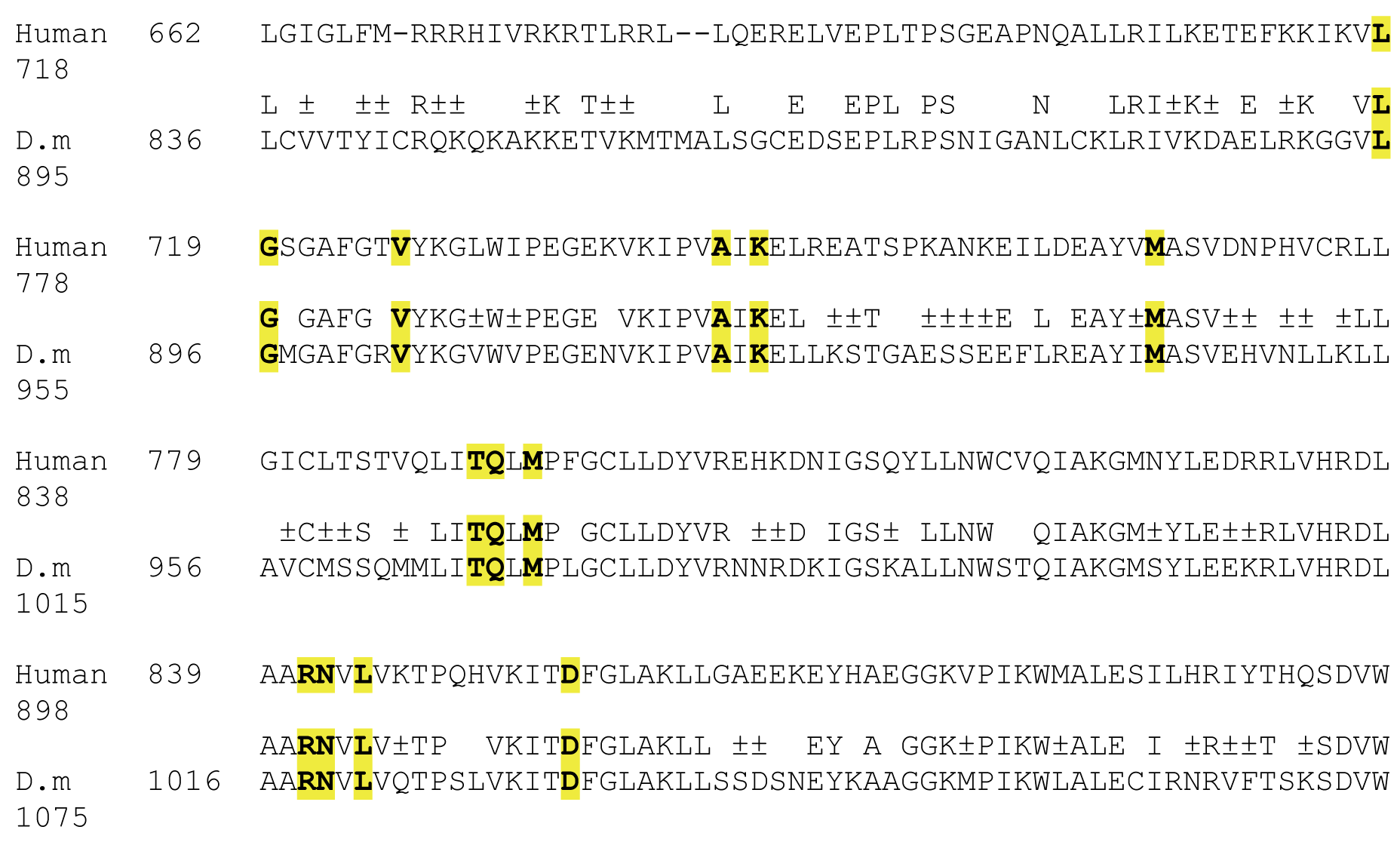

Supplement: Figure S1 — Sequence alignment of the EGFR proteins from human and Drosophila melanogaster. (TIF) [file pone.0029782.s001.tif]

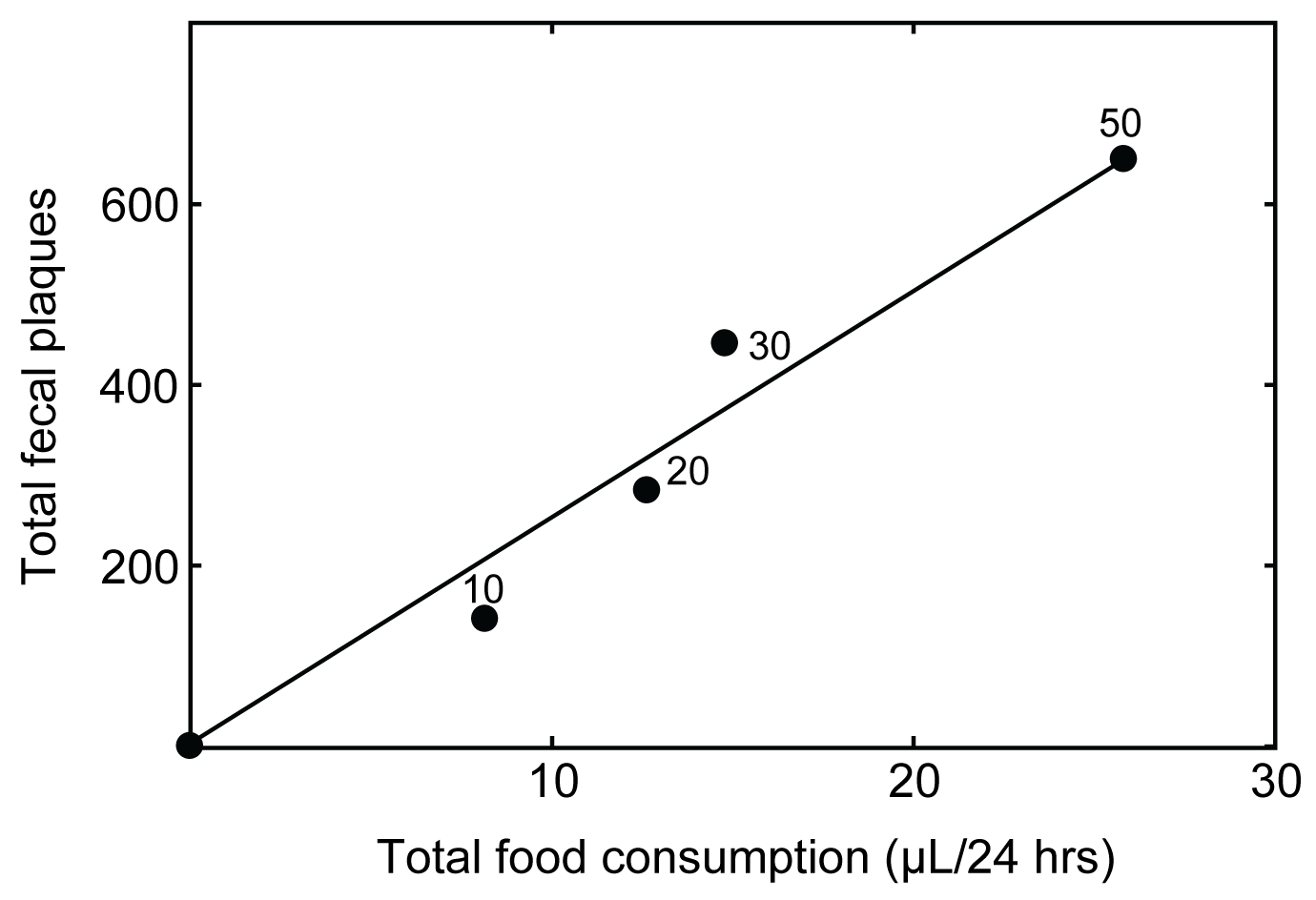

Supplement: Figure S2 — There is a strong correlation between food consumption measured by the CAFE and FPAs. (TIF) [file pone.0029782.s002.tif]

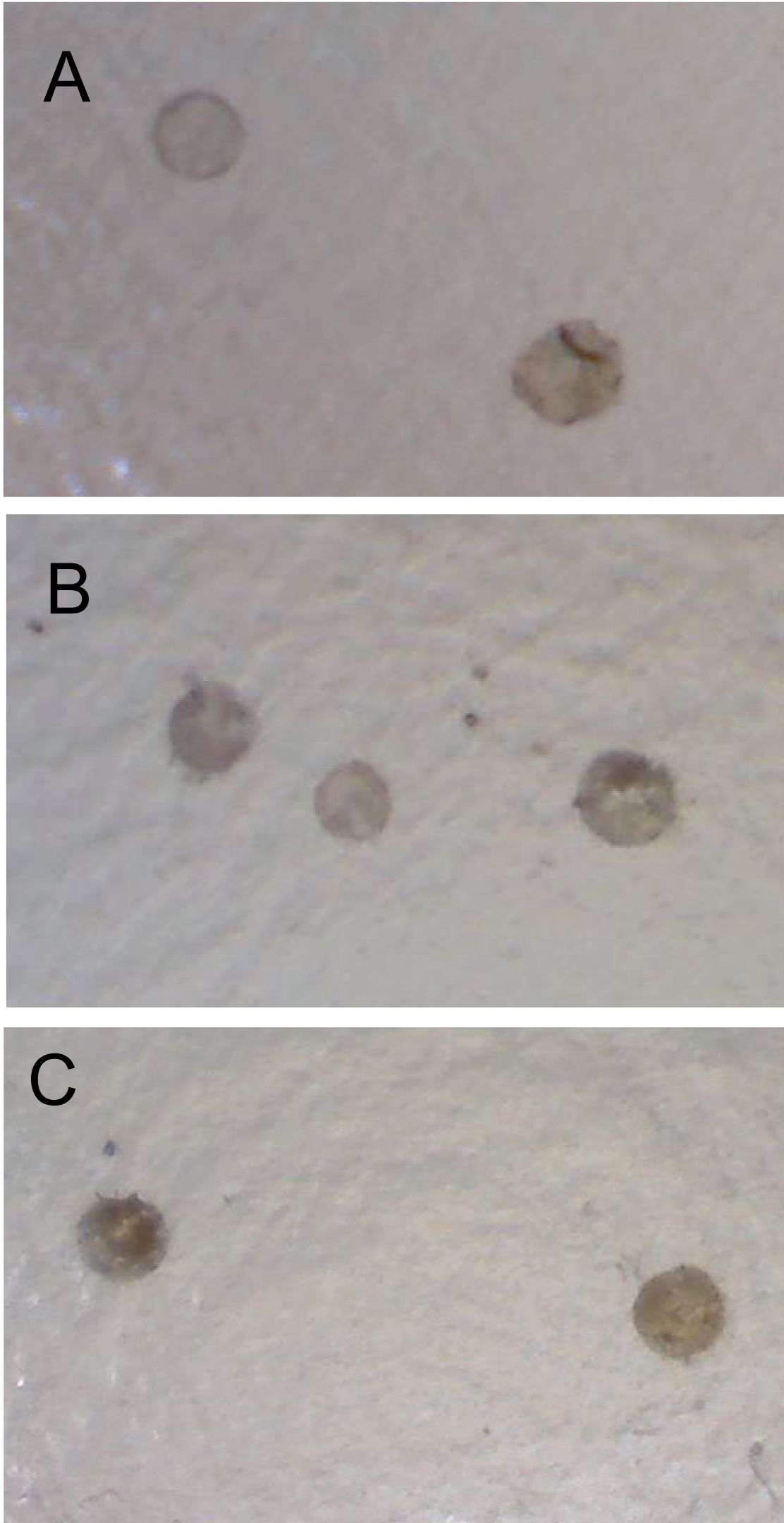

Supplement: Figure S3 — Representative plaques from control and drug treated Drosophila. (TIF) [file pone.0029782.s003.tif]

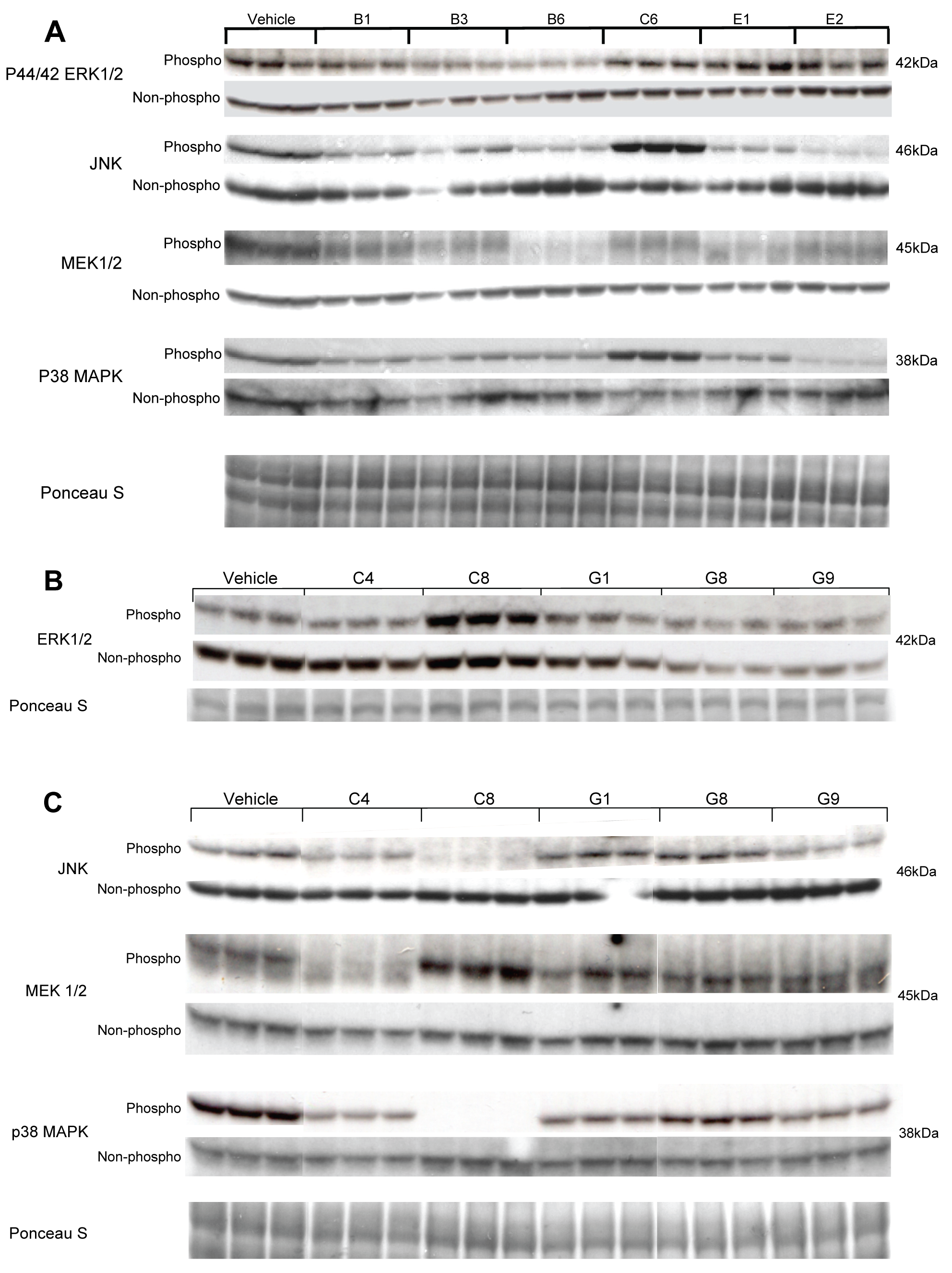

Supplement: Figure S4 — Representative Western blot results of the effects of protein kinase inhibitors on intracellular signaling in Drosophila S2 cells. (TIF) [file pone.0029782.s004.tif]

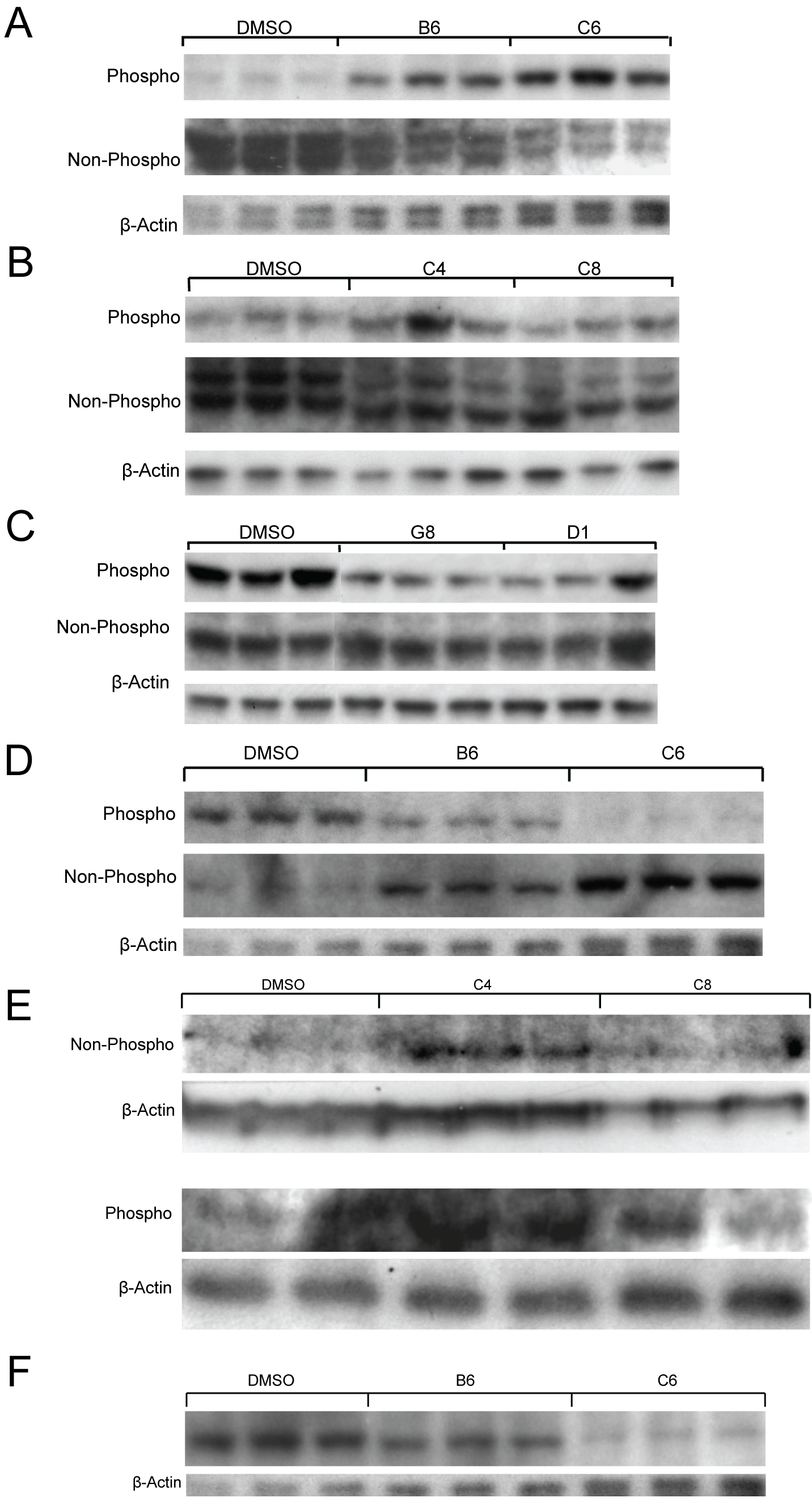

Supplement: Figure S5 — Representative Western blot results of the effects of the protein kinase inhibitors indicated at the top each figure on intracellular signaling in intact Drosophila. (TIF) [file pone.0029782.s005.tif]

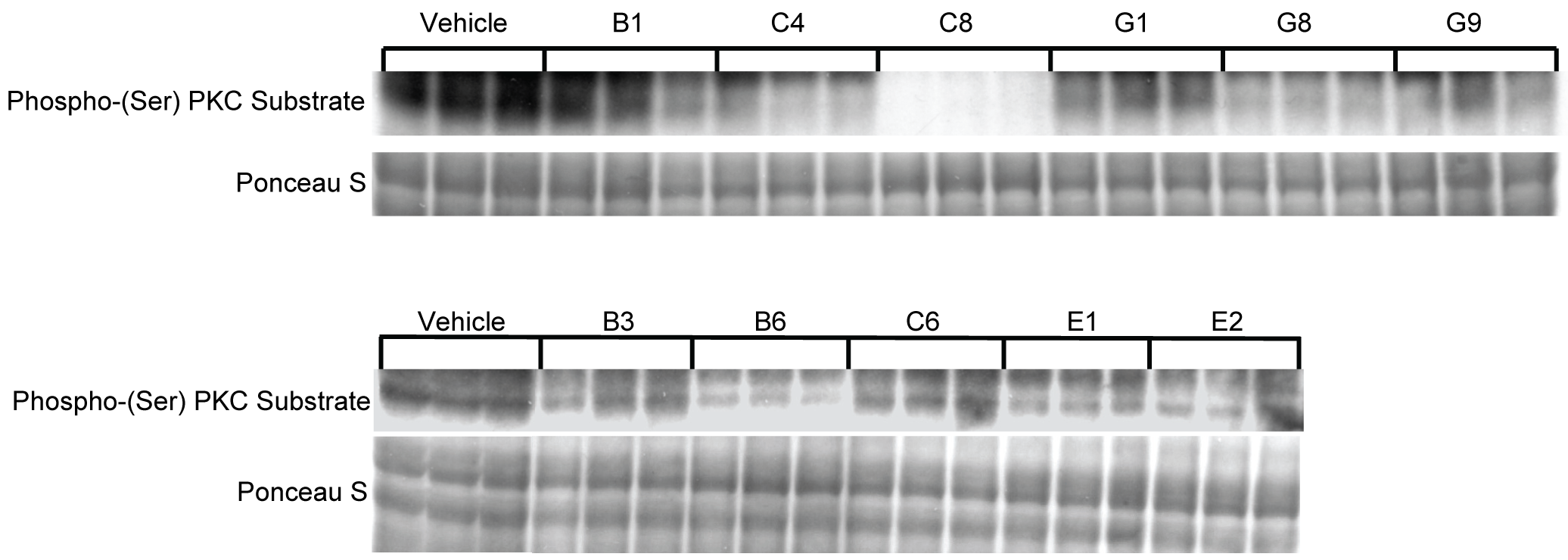

Supplement: Figure S6 — Representative PKC-substrate serine phosphorylation measured using an antibody specific for phospho-(Ser) PKC substrates. (TIF) [file pone.0029782.s006.tif]

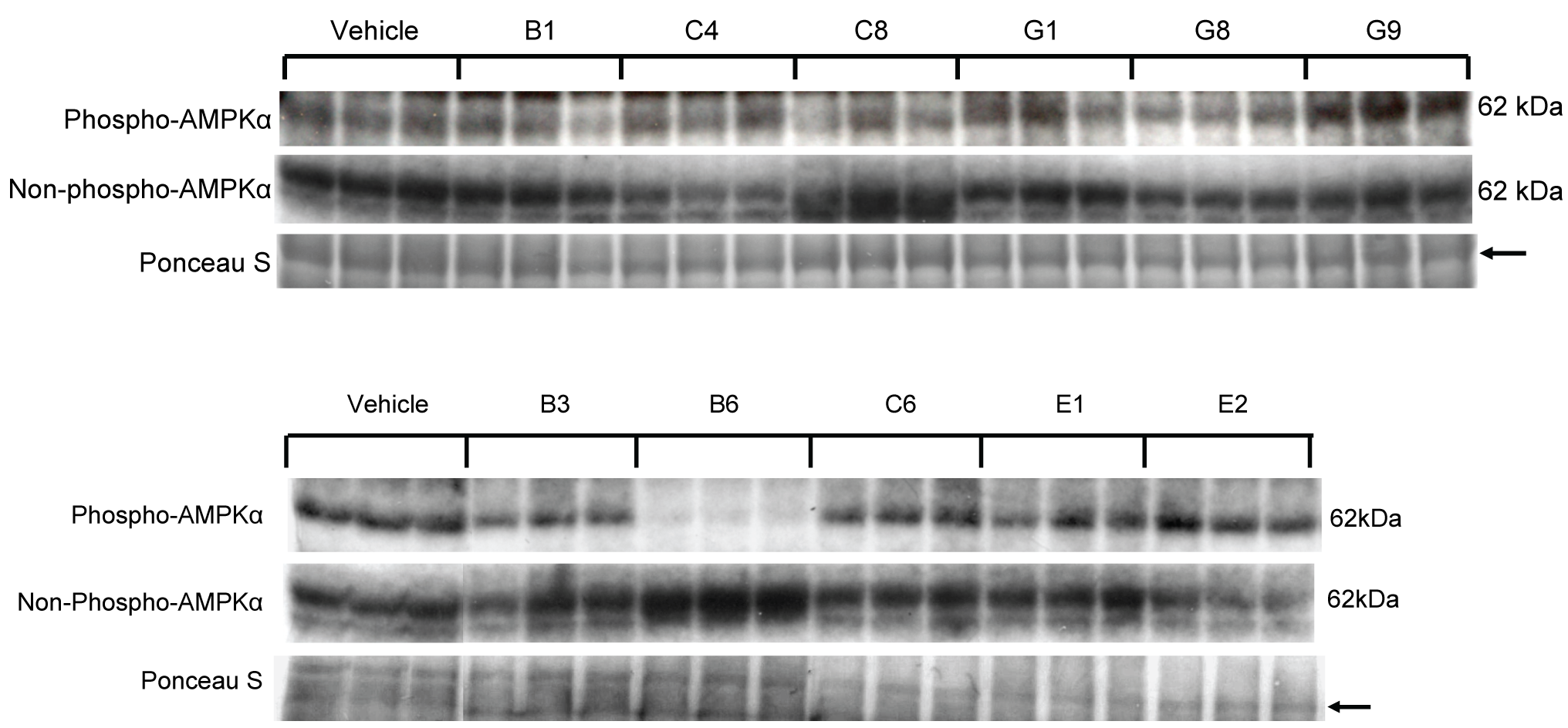

Supplement: Figure S7 — The results of representative Western blots probed with antibodies directed against the phosphorylated and non-phosphorylated forms of AMPKα. (TIF) [file pone.0029782.s007.tif]

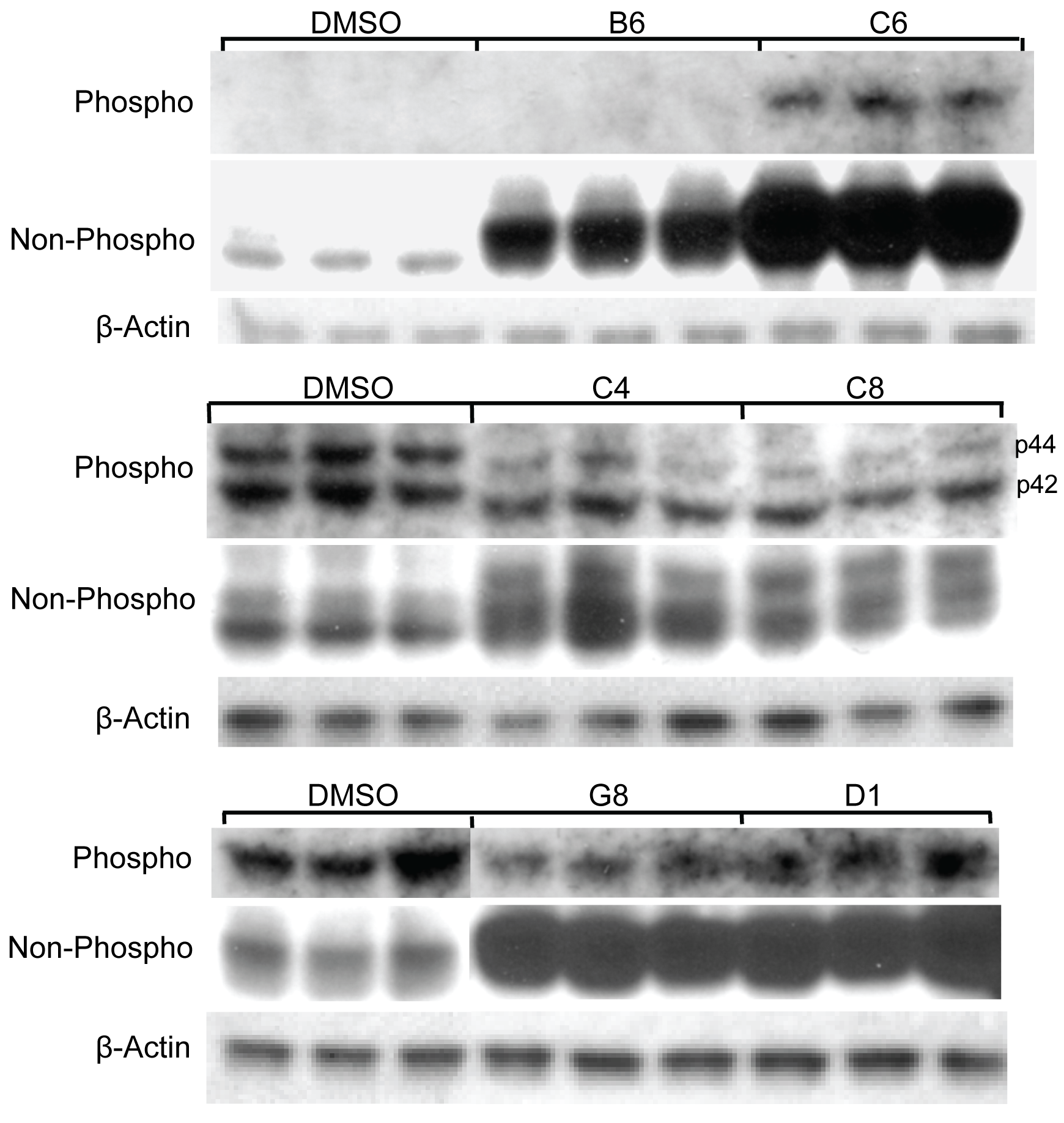

Supplement: Figure S8 — The results of representative Western blots probed with antibodies directed against the phosphorylated or non-phosphorylated forms of ERK1/2 or β-actin. (TIF) [file pone.0029782.s008.tif]
