# Supplementary material for: Novel Protein Kinase Signaling Systems Regulating Lifespan Identified by Small Molecule Library Screening Using Drosophila
Source: PLoS One. 2012 Feb 20;7(2):e29782. doi: 10.1371/journal.pone.0029782 (PMC3282711; doi:10.1371/journal.pone.0029782)
Supplement: Table S1 — Comprehensive list of the compounds present in the BioMol kinase inhibitor library. (DOC) [file pone.0029782.s009.doc]

**Table S1.**  Comprehensive list of the compounds present in the BioMol kinase inhibitor library.

**BioMol**

**ID No. Drug Name**

B1 PD-98059

B2 U-0126

B3 SB-203580

B4 H-7

B5 H-9

B6 Staurosporine

B7 AG-494

B8 AG-825

B9 Lavendustin A

B10 RG-14620

B11 Tyrphostin 23

B12 Tyrphostin 25

C1 Tyrphostin 46

C2 Tyrphostin 47

C3 Tyrphostin 51

C4 Tyrphostin 1

C5 Tyrphostin AG 1288

C6 Tyrphostin AG 1478

C7 Tyrphostin AG 1295

C8 Tyrphostin 9

C9 HNMPA (Hydroxy-2-
 naphthalenylmethylphosphonic
 acid)

C10 PKC-412

C11 Piceatannol

C12 PP1

D1 AG-490

D2 AG-126

D3 AG-370

D4 AG-879

D5 LY 294002

D6 Wortmannin

D7 GF 109203X

D8 Hypericin

D9 Ro 31-8220

D10 Sphingosine

D11 H-89

D12 H-8

E1 HA-1004

E2 HA-1077

E3 HDBA (2-Hydroxy-5-(2,5-
 dihydroxybenzylamino)benzoic
 acid)

E4 KN-62

**BioMol**

**ID No. Drug Name**

E5 KN-93

E6 ML-7

E7 ML-9

E8 2-Aminopurine

E9 N9-Isopropyl-olomoucine

E10 Olomoucine

E11 iso-Olomoucine

E12 Roscovitine

F1 5-Iodotubercidin

F2 LFM-A13

F3 SB-202190

F4 PP2

F5 ZM 336372

F6 SU 4312

F7 AG-1296

F8 GW 5074

F9 Palmitoyl-DL-carnitine Cl

F10 Rottlerin

F11 Genistein

F12 Daidzein

G1 Erbstatin analog

G2 Quercetin dihydrate

G3 SU1498

G4 ZM 449829

G5 BAY 11-7082

G6 DRB (5,6-Dichloro-1-b-D-
 ribofuranosylbenzimidazole)

G7 HBDDE (2,2',3,3',4,4'-
 Hexahydroxy-1,1'-biphenyl-6,6'-
 dimethanol dimethyl ether)

G8 SP 600125

G9 Indirubin

G10 Indirubin-3'-monoxime

G11 Y-27632

G12 Kenpaullone GSK-3b

H1 Terreic acid

H2 Triciribine

H3 BML-257

H4 SC-514

H5 BML-259 H6 Apigenin

H7 BML-265 (Erlotinib analog)

H8 Rapamycin (Everolimus was
 used for the studies reported here.)
