# Supplementary material for: Novel Protein Kinase Signaling Systems Regulating Lifespan Identified by Small Molecule Library Screening Using Drosophila
Source: PLoS One. 2012 Feb 20;7(2):e29782. doi: 10.1371/journal.pone.0029782 (PMC3282711; doi:10.1371/journal.pone.0029782)
Supplement: Table S2 — Reported targets of the protein kinase inhibitors confirmed to extend Drosophila lifespan. (DOC) [file pone.0029782.s010.doc]

**Table S2.** Reported targets of the protein kinase inhibitors confirmed to extend *Drosophila* lifespan.

**Biomol**

**ID# Name Major target(s)**

B1 PD98059 **MEK1**^a^ & 2 (IC_50_ 4 μM & 50 μM) [1]. Blocks the activation of the MAPK cascade above MAPK kinase (MKK1).

B3 SB203580 **p38MAPK** (IC_50_ = 34-38 nM), **RICK** (IC_50_ = 16 nM), and **CK1** and **GAK** (IC_50_ ≈ 114 nM)[1,2].

B6 Staurosporine **PKC** (IC_50_ 0.7 nM), **PKA**, **PKG**, **CaMKII** and **MLCK** (IC_50_ 1-20 nM) [3]. Much lower affinity inhibitor of PI3K (IC_50_ 9 µM) [4].

C4 Tyrphostin 1 **EGFR** (IC_50_ 1,250 μM) [5-9]. May inhibit an unidentified tyrosine kinase [10-12].

C6 Tyrphostin **EGFR** (IC_50_ 3 nM) [13]. A much lower affinity inhibitor

AG1478 of Kv1.5 potassium channels (IC50 = 9.8 µM) [14]. Supresses Erk1/2 activation, inhibits FOS gene expression [8,15].

C7 Tyrphostin **PDGFR** (IC_50_ 500 nM) [16].

AG1295

C8 Tyrphostin 9 **PDGFR** (IC50=1.2 μM) [6]. Also an uncoupler of oxidative

(AG17) phosphorylation (10 nM), which disrupts mitochondrial structure and function [17].

D1 AG-490 **EGFR** (IC_50_ 100 nM). A much lower affinity inhibitor of JAK2 (IC_50_ 56.8μM). Displays apoptotic and antiproliferative properties with IC_50_ of 1.7, 2.8 and 6.1 µM in 2E8, Baf/3 and Jurkat cells, respectively [18,19].

E1 HA-1004 **PKG** (Ki 1.3 μM), **PKA** (Ki 2.3 μM), **CaMKII** (Ki = 13 μM); and with lower potency **PKC** (Ki 40 μM) and **MLCK** (Ki 150 μM) [20].

E2 HA-1077 **ROCK I & II** (1.9 µM for ROCK II), **PRK2** (4 µM); **MSK1** (5 µM);

(a.k.a. fasudil) **MAPKAP-K1b** (19 uM); **PHK** (44 µM) [1]. ROCK inhibition may mimic the "pleotropic" effects of statins. ROCK inhibition reduces blood vessel constriction, decreases pulmonary arterial pressure, inhibits tumor angiogenesis and metastasis, and improves insulin signaling in rodents [1,21].

E5 KN-93 **CaMKII** (IC50 0.37 μM) [22]. Direct extracellular open channel blocker of **voltage-gated** **potassium channels** (IC50 = 307 nM for Kv1.5). This activity is independent of the CaM kinase II inhibition [23].

F4 PP2 AG1879 (a.k.a. pyrazolopyrimidine 2; PP2; AG 1879) **Src family of kinases** [p56^lck^ (IC_50_ 4 nM), p59^fyn^T (a.k.a. FYN(B); IC_50_ 5 nM), Hck (IC_50_ 5 nM), and Src (IC_50_ 100 nM)] [24,25]. Does not significantly inhibit EGFR kinase (IC_50_ = 480 nM), CSK (IC_50_ = 730 nM), JAK2 (IC_50_ > 50 μM), or ZAP-70 (IC_50_ >100 μM) .

G1 Erbstatin analog **EGFR** kinase activity (IC_50_ = 770 nM) [26,27].

G2 Quercetin **PIM1** kinase (PIM1K) (IC_50_ = 43 **nM**; ED_50_ 5.5 μM); **phospholipase A2**

dihydrate (IC_50_ = 2 μM) [4] and **PI3K** (IC_50_ 3.8 μM) [28]. It inhibits **AMPK**, **CK2**, **MAPKAP-K1/RSK2** and **S6K1** with a potency similar to that for **PI3K** [1].

G8 SP600125 **JNK** (IC_50_=40 nM for JNK-1 and JNK-2 and 90 nM for JNK-3) [29]. 300-fold greater selectivity for JNK versus ERK1 and p38MAPK. Inhibits the phosphorylation of c-Jun and blocks the expression of IL-2, IFN-γ, TNF-α, and COX-2 [29].

G9 Indirubin **GSK-3β** (IC_50_ 0.6 µM) and **CDK5** & **CDK1** (IC_50_ 5.5 & 10 µM) [30].

H8 Everolimus **TOR** (mTORC1; mammalian TOR complex 1) (IC_50_ 2 nM) [31]

^a^Abbreviations used: **AMPK**, AMP-activated protein kinase; **CaMKII**, Ca^2±^/calmodulin-dependent protein kinase IIα; **CDK1 and 5**, cyclin-dependent kinase 1 and 5; **CK1 and 2**, casein kinase 1; **CSK1/2**, human C-terminal Src kinase; **EGFR**, epidermal growth factor receptor [a subfamily of four closely related receptor tyrosine kinases EGFR (ErbB-1), HER2/c-neu (ErbB-2), Her 3 (ErbB-3) and Her 4 (ErbB-4)]; **Erk1/2**, extracellular-signal-regulated kinases 1 and 2 (p44 and p42 mitogen activated protein kinase); **JAK2**, Janus kinase 2; **JNK**, c-Jun N-terminal kinases; **GAK**, cyclin G-associated kinase, **GSK-3β**, glycogen synthase kinase 3 beta (a.k.a. GSK3B); **MLCK**, mycin light chain kinase; **MAPK**, mitogen activated protein kinase; **MAPKAP-K1bp**, mitogen activated protein kinase-activated protein kinase 1b; **MEK**, mitogen-activated protein kinase kinase 1 (a.k.a. MKK, MAPKK, MAPK/ERK kinase); **MSK1**, mitogen- and stress-activated protein kinase; **p38MAPK**, p38 mitogen-activated protein kinase (a.k.a. SAPK2a/p38; mitogen-activated protein kinase p38α, mitogen-activated protein kinase 14); **PDGFR**, platelet-derived growth factor receptor; **PHK**, phosphorylase kinase; **PIM1**, proto-oncogene serine/threonine-protein kinase; **PKA**, protein kinase, cAMP-dependent, catalytic, β1 (a.k.a. cAMP-dependent protein kinase catalytic β subunit isoform 4ab); **PKC**, protein kinase C (a.k.a. PKCα type); **PI3K**, phosphatidylinositol 3' kinase; **PKG**, cGMP-dependent protein kinase; **PRK2**, protein kinase C-related kinase 2; **RICK**, Rip-like interacting caspase-like apoptosis-regulatory protein kinase; **ROCK I & II**, Rho dependent kinases I and II; **S6K1**, ribosomal S6 protein kinase 1; **Src family of kinases**, v-src sarcoma (Schmidt-Ruppin A-2) viral oncogene homolog family; **TOR**, target of rapamycin.

**Supporting Table 2 References**

1. Davies SP, Reddy H, Caivano M, Cohen P (2000) Specificity and mechanism of action of some commonly used protein kinase inhibitors. Biochem J 351:95-105.

2. Godl K, Wissing J, Kurtenbach A, Habenberger P, Blencke S, et al. (2003) An efficient proteomics method to identify the cellular targets of protein kinase inhibitors. Proc Natl Acad Sci U S A 100:15434-15439.

3. Ruegg UT, Burgess GM (1989) Staurosporine, K-252 and UCN-01: potent but nonspecific inhibitors of protein kinases. Trends Pharmacol Sci 10:218-220.

4. Walker EH, Pacold ME, Perisic O, Stephens L, Hawkins PT, et al. (2000) Structural determinants of phosphoinositide 3-kinase inhibition by wortmannin, LY294002, quercetin, myricetin, and staurosporine. Mol Cell 6:909-919.

5. Pumiglia KM, Lau LF, Huang CK, Burroughs S, Feinstein MB (1992) Activation of signal transduction in platelets by the tyrosine phosphatase inhibitor pervanadate (vanadyl hydroperoxide). Biochem J 286:441-449.

6. Liu M, Liu J, Buch S, Tanswell AK, Post M (1995) Antisense oligonucleotides for PDGF-B and its receptor inhibit mechanical strain-induced fetal lung cell growth. Am J Physiol 269:L178-L184.

7. Munaron L, Fiorio PA (2000) Calcium influx induced by activation of tyrosine kinase receptors in cultured bovine aortic endothelial cells. J Cell Physiol 185:454-463.

8. Tsukagoshi H, Kawata T, Shimizu Y, Ishizuka T, Dobashi K, et al. (2002) 4-Hydroxy-2-nonenal enhances fibronectin production by IMR-90 human lung fibroblasts partly via activation of epidermal growth factor receptor-linked extracellular signal-regulated kinase p44/42 pathway. Toxicol Appl Pharmacol 184:127-135.

9. Sugano E, Tomita H, Ishiguro S, Abe T, Tamai M (2005) Establishment of effective methods for transducing genes into iris pigment epithelial cells by using adeno-associated virus type 2. Invest Ophthalmol Vis Sci 46:3341-3348.

10. Wijetunge S, Aalkjaer C, Schachter M, Hughes AD (1992) Tyrosine kinase inhibitors block calcium channel currents in vascular smooth muscle cells. Biochem Biophys Res Commun 189:1620-1623.

11. Standley PR, Obards TJ, Martina CL (1999) Cyclic stretch regulates autocrine IGF-I in vascular smooth muscle cells: implications in vascular hyperplasia. Am J Physiol 276:E697-E705.

12. Smith AD, Collaco RF, Trempe JP (2003) Enhancement of recombinant adeno-associated virus type 2-mediated transgene expression in a lung epithelial cell line by inhibition of the epidermal growth factor receptor. J Virol 77:6394-6404.

13. Levitzki A, Gazit A (1995) Tyrosine kinase inhibition: an approach to drug development. Science 267:1782-1788.

14. Choi BH, Choi JS, Rhie DJ, Yoon SH, Min DS, et al. (2002) Direct inhibition of the cloned Kv1.5 channel by AG-1478, a tyrosine kinase inhibitor. Am J Physiol Cell Physiol 282:C1461-C1468.

15. Daub H, Weiss FU, Wallasch C, Ullrich A (1996) Role of transactivation of the EGF receptor in signalling by G-protein-coupled receptors. Nature 379:557-560.

16. Kovalenko M, Gazit A, Bohmer A, Rorsman C, Ronnstrand L, et al. (1994) Selective platelet-derived growth factor receptor kinase blockers reverse sis-transformation. Cancer Res 54:6106-6114.

17. Liang BC, Ullyatt E (1998) Chemosensitization of glioblastoma cells to bis-dichloroethyl-nitrosourea with tyrphostin AG17. Clin Cancer Res 4:773-781.

18. Wang LH, Kirken RA, Erwin RA, Yu CR, Farrar WL (1999) JAK3, STAT, and MAPK signaling pathways as novel molecular targets for the tyrphostin AG-490 regulation of IL-2-mediated T cell response. J Immunol 162:3897-3904.

19. Meydan N, Grunberger T, Dadi H, Shahar M, Arpaia E, et al. (1996) Inhibition of acute lymphoblastic leukaemia by a Jak-2 inhibitor. Nature 379:645-648.

20. Hidaka H, Inagaki M, Kawamoto S, Sasaki Y (1984) Isoquinolinesulfonamides, novel and potent inhibitors of cyclic nucleotide dependent protein kinase and protein kinase C. Biochemistry 23:5036-5041.

21. Olson MF (2008) Applications for ROCK kinase inhibition. Curr Opin Cell Biol 20:242-248.

22. Tokumitsu H, Chijiwa T, Hagiwara M, Mizutani A, Terasawa M, et al. (1990) KN-62, 1-[N,O-bis(5-isoquinolinesulfonyl)-N-methyl-L-tyrosyl]-4-phenylpiperazi ne, a specific inhibitor of Ca2+/calmodulin-dependent protein kinase II. J Biol Chem 265:4315-4320.

23. Rezazadeh S, Claydon TW, Fedida D (2006) KN-93 (2-[N-(2-hydroxyethyl)]-N-(4-methoxybenzenesulfonyl)]amino-N-(4-chlorocinnamyl)-N -methylbenzylamine), a calcium/calmodulin-dependent protein kinase II inhibitor, is a direct extracellular blocker of voltage-gated potassium channels. J Pharmacol Exp Ther 317:292-299.

24. Hanke JH, Gardner JP, Dow RL, Changelian PS, Brissette WH, et al. (1996) Discovery of a novel, potent, and Src family-selective tyrosine kinase inhibitor. Study of Lck- and FynT-dependent T cell activation. J Biol Chem 271:695-701.

25. Bain J, McLauchlan H, Elliott M, Cohen P (2003) The specificities of protein kinase inhibitors: an update. Biochem J 371:199-204.

26. Umezawa K, Sugata D, Yamashita K, Johtoh N, Shibuya M (1992) Inhibition of epidermal growth factor receptor functions by tyrosine kinase inhibitors in NIH3T3 cells. FEBS Lett 314:289-292.

27. Umezawa K, Hori T, Tajima H, Imoto M, Isshiki K, et al. (1990) Inhibition of epidermal growth factor-induced DNA synthesis by tyrosine kinase inhibitors. FEBS Lett 260:198-200.

28. Holder S, Zemskova M, Zhang C, Tabrizizad M, Bremer R, et al. (2007) Characterization of a potent and selective small-molecule inhibitor of the PIM1 kinase. Mol Cancer Ther 6:163-172.

29. Bennett BL, Sasaki DT, Murray BW, O'Leary EC, Sakata ST, et al. (2001) SP600125, an anthrapyrazolone inhibitor of Jun N-terminal kinase. Proc Natl Acad Sci U S A 98:13681-13686.

30. Leclerc S, Garnier M, Hoessel R, Marko D, Bibb JA, et al. (2001) Indirubins inhibit glycogen synthase kinase-3 beta and CDK5/p25, two protein kinases involved in abnormal tau phosphorylation in Alzheimer's disease. A property common to most cyclin-dependent kinase inhibitors? J Biol Chem 276:251-260.

31. Beuvink I, Boulay A, Fumagalli S, Zilbermann F, Ruetz S, et al. (2005) The mTOR inhibitor RAD001 sensitizes tumor cells to DNA-damaged induced apoptosis through inhibition of p21 translation. Cell 120:747-759.
