# Supplementary material for: Novel Protein Kinase Signaling Systems Regulating Lifespan Identified by Small Molecule Library Screening Using Drosophila
Source: PLoS One. 2012 Feb 20;7(2):e29782. doi: 10.1371/journal.pone.0029782 (PMC3282711; doi:10.1371/journal.pone.0029782)
Supplement: Table S3 — Summary of the pathways regulating Drosophila lifespan identified through these studies. (DOC) [file pone.0029782.s011.doc]

**Table S3.** Summary of the pathways regulating *Drosophila* lifespan identified through these studies.

**Biomol Compound**

**ID# names Major target(s)^a^ Major Pathway(s) in adult *Drosophila* (abbreviated)**

B1 PD-98059 MEK^b^ RTK🡪Ras (*Ras85D*)^b^🡪Raf (*pole hold*)🡪MEKKà**MEK** (*rolled*)🡪
 ERKàCell proliferation/Survival/Differentiation [1]

B3 SB203580 p38MAPK Stress🡪GPCR/Damage/TGFβ🡪**p38MAPKa/b/c**🡪Stress response; Innate
 [*licorne (lic)*] immunity/inflammation [2]
 RICK TLR2/ILR/NOD🡪**RICK**/Rip2🡪TAK1🡪NFκB/JNK🡪cytokine
 production/cell proliferation/innate immunity
 [3]
 CK1 Wnt🡪Fz🡪Dsh🡪**CK1**🡪GSK3🡪β-catenin🡪LEF/TCF🡪circadian
 rhythm/cell proliferation & fate [4]
 GAK **GAK**🡪clathrin-mediated membrane trafficking/mitotic progression
 [5]

B6 Staurosporine PKA GPCR🡪cAMP🡪**PKA**🡪Reduced age-related memory impairment
 [6]

PKC GPCR🡪DAG/Ca^2±^🡪**PKCα**🡪MLCK🡪Endothelial cell contractile force
 generation [7]

PKG NOS🡪NO🡪sGC🡪cGMP🡪**PKG**🡪Visual pattern memory, foraging
 behavior, anoxic/thermal tolerance [8-11]

CaMKII GPCR/RTK🡪DAG/Ca^2±^🡪**CaMKII**🡪PI3K&MLCK🡪Learning (synaptic
 connections&axon pathway formation)/neuromuscular junctions
 contractile force [12,13]

MLCK **MLCK**🡪MRLC🡪cytoskeletal contractility [14]

PI3K Insulin/RTK🡪**PI3K**🡪Akt🡪TOR🡪GSK3àCell growth/cell cycle

progression/metabolism/synaptic plasticity [13,15,16]

C4 Tyrphostin 1 Unknown/ **EGFR** (*Torpedo*)àRas (*Ras85D*)🡪Raf (*pole hold*)🡪MEKKàMEK
 EGFR (*rolled*)🡪ERKàCell proliferation/survival/differentiation
 [1]

C6 Tyrphostin EGFR **EGFR** (*Torpedo*)àRas (*Ras85D*)🡪Raf (*pole hold*)🡪MEKKàMEK
 AG1478 (*rolled*)🡪ErkàCell proliferation/Survival/Differentiation
 [1]

C7 Tyrphostin PDGFR **PDGFR/VEGFR** (Pvr)àGrb2-SosàRas🡪ErkàCell proliferation/
 AG1295 Migration/Survival/Differentiation, Innate immunity, development
 [17-24].

C8 Tyrphostin 9 PDGFR **PDGFR/VEGFR** (Pvr)àGrb2-SosàRas🡪ErkàCell proliferation/
 Migration/Survival/Differentiation, Innate immunity, development
 [17-24].

D1 AG-490 JAK2 **JAK**🡪Stat🡪MEK/TOR/FOXOàStress response/Innate immunity
 [25,26]

E1 HA-1004 PKA/ GPCR🡪cAMP🡪**PKA**🡪Reduced age-related memory impairment
 PKG [6]
 NOS🡪NO🡪sGC🡪cGMP🡪**PKG**🡪Visual pattern memory, foraging
 behavior, anoxic/thermal tolerance [8-11]

E2 HA-1077 ROCK Rho🡪**ROCK**I/IIàMLCK🡪MRLC🡪stress fiber formation/cytoskeletal
 contractile force generation [27]

E5 KN-93 CaMKII GPCR/RTK🡪DAG/Ca^2±^🡪**CaMKII**🡪PI3K&MLCK🡪Learning
 (synaptic connections&axon pathway formation)/neuromuscular
 junctions contractile force [12,13]

F4 PP2 AG1879 Src RTKà**Src**(Src42A, Src64B)àAbl/Stat3/Rac🡪Myc🡪Cell growth/
 Division/Invasion [28,29].
 PDGFR is directly activated by Src family kinases [29].

G1 Erbstatin EGFR **EGFR**àRas (*Ras85D*)🡪Raf (pole hold)🡪MEKKàMEK (*rolled*)🡪

analog ERKàCell proliferation/survival/differentiation [1]

G2 Quercetin PI3K Insulin/RTK🡪**PI3K**🡪Akt🡪TOR🡪GSK3àCell growth/cell cycle
 dihydrate progression/metabolism/synaptic plasticity [13,15,16]

G8 SP600125 JNK Stress/inflammation🡪**JNK**àIntestinal stem cell proliferation🡪dysplasia
 [30].

G9 Indirubin GSK3 Insulin/RTK🡪PI3KàAkt🡪TOR🡪**GSK3**àCell growth/cell cycle
 progression/metabolism/synaptic plasticity [13,15,16]

H8 Everolimus TOR Insulin/RTKàPI3KàAktà**TOR**🡪GSK3àCell growth/cell cycle
 progression/metabolism/synaptic plasticity [13,15,16]

^a^Abbreviations used: **Akt** (a.k.a. Protein Kinase B), murine thymoma viral oncogene homolog 1; **CaMKII**, Ca^++^/calmodulin-dependent protein kinase IIα; **CK1**, casein kinase 1; **Dsh**, dishevelled; **EGFR**, epidermal growth factor receptor kinase; Fz, Frizzled; **GAK**, cyclin G associated kinase; **GSK3**, Glycogen synthase kinase 3β; **ILR**, interlukin-1 receptor; **JAK**, Janus kinase; **JNK**, c-Jun N-terminal kinases; **LEF**, lymphoid enhancer-binding factor; **MEK**, mitogen-activated protein kinase kinase 1 (a.k.a. MKK, MAPKK, MAPK/ERK kinase); **MLCK**, myosin light-chain kinase; **MRLC**, myosin regulatory light chain; **NOD**, nucleotide-binding oligomerization domain containing; **p38MAPK**, p38 mitogen-activated protein kinase (a.k.a. p38, Mitogen-activated protein kinase p38α, mitogen-activated protein kinase 14); **PDGFR/VEGFR**, platelet-derived growth factor receptor/vascular endothelial growth factor receptor; **PI3K**, phosphatidylinositol 3-kinase; **PKA**, protein kinase, cAMP-dependent, catalytic, β1 (a.k.a. cAMP-dependent protein kinase catalytic β subunit isoform 4ab); **PKC**, protein kinase C (a.k.a., PKCα type); **PKG**, cGMP-dependent protein kinase; **RICK** (a.k.a. RIP2), receptor-interacting serine-threonine kinase 2; **RTK**, receptor tyrosine kinases; **ROCKs**, Rho-GTPase effector kinases I and II; **sGC**, soluble guanylate cyclase; **Src**, v-src sarcoma (Schmidt-Ruppin A-2) viral oncogene homolog; **TAK1**, mitogen-activated protein kinase kinase kinase 7; **TCF**; T-cell specific, HMG-box transcription factor; **TLR**, Toll-like receptor 2; **TOR**, target of rapamycin.

^b^The names in parenthese are the *Drosophila* homologues of the mammalian proteins.

**Supporting Table 3 References**

1. Buchon N, Broderick NA, Kuraishi T, Lemaitre B (2010) Drosophila EGFR pathway coordinates stem cell proliferation and gut remodeling following infection. BMC Biol 8:152.:152.

2. Chen J, Xie C, Tian L, Hong L, Wu X, et al. (2010) Participation of the p38 pathway in Drosophila host defense against pathogenic bacteria and fungi. Proc Natl Acad Sci U S A 107:20774-20779.

3. Kobayashi K, Inohara N, Hernandez LD, Galan JE, Nunez G, et al. (2002) RICK/Rip2/CARDIAK mediates signalling for receptors of the innate and adaptive immune systems. Nature 416:194-199.

4. Davidson G (2010) The cell cycle and Wnt. Cell Cycle 9:1667-1668.

5. Shimizu H, Nagamori I, Yabuta N, Nojima H (2009) GAK, a regulator of clathrin-mediated membrane traffic, also controls centrosome integrity and chromosome congression. J Cell Sci 122:3145-3152.

6. Yamazaki D, Horiuchi J, Miyashita T, Saitoe M (2010) Acute inhibition of PKA activity at old ages ameliorates age-related memory impairment in Drosophila. J Neurosci 30:15573-15577.

7. Mizuno K, Noda K, Ueda Y, Hanaki H, Saido TC, et al. (1995) UCN-01, an anti-tumor drug, is a selective inhibitor of the conventional PKC subfamily. FEBS Lett 359:259-261.

8. Hou Q, Jiang H, Zhang X, Guo C, Huang B, et al. (2011) Nitric oxide metabolism controlled by formaldehyde dehydrogenase (fdh, homolog of mammalian GSNOR) plays a crucial role in visual pattern memory in Drosophila. Nitric Oxide 24:17-24.

9. Kaun KR, Sokolowski MB (2009) cGMP-dependent protein kinase: linking foraging to energy homeostasis. Genome 52:1-7.

10. Dawson-Scully K, Armstrong GA, Kent C, Robertson RM, Sokolowski MB (2007) Natural variation in the thermotolerance of neural function and behavior due to a cGMP-dependent protein kinase. PLoS ONE 2:e773.

11. Dawson-Scully K, Bukvic D, Chakaborty-Chatterjee M, Ferreira R, Milton SL, et al. (2010) Controlling anoxic tolerance in adult Drosophila via the cGMP-PKG pathway. J Exp Biol 213:2410-2416.

12. Carrillo RA, Olsen DP, Yoon KS, Keshishian H (2010) Presynaptic activity and CaMKII modulate retrograde semaphorin signaling and synaptic refinement. Neuron 68:32-44.

13. Chun-Jen Lin C, Summerville JB, Howlett E, Stern M (2011) The Metabotropic Glutamate Receptor Activates the Lipid Kinase PI3K in Drosophila Motor Neurons through the Calcium/Calmodulin-dependent Protein Kinase II (CaMKII) and the Non-receptor Tyrosine Protein Kinase DFak. Genetics 188:601-613.

14. Takashima S (2009) Phosphorylation of myosin regulatory light chain by myosin light chain kinase, and muscle contraction. Circ J 73:208-213.

15. Powis KV, Macdougall LK (2011) The localisation of PtdIns3P in Drosophila fat responds to nutrients but not insulin: a role for Class III but not Class II phosphoinositide 3-kinases. Cell Signal 23:1153-1161.

16. Willecke M, Toggweiler J, Basler K (2011) Loss of PI3K blocks cell-cycle progression in a Drosophila tumor model. Oncogene 30:4067-4074.

17. Cho NK, Keyes L, Johnson E, Heller J, Ryner L, et al. (2002) Developmental control of blood cell migration by the Drosophila VEGF pathway. Cell 108:865-876.

18. Bianco A, Poukkula M, Cliffe A, Mathieu J, Luque CM, et al. (2007) Two distinct modes of guidance signalling during collective migration of border cells. Nature 448:362-365.

19. Duchek P, Somogyi K, Jekely G, Beccari S, Rorth P (2001) Guidance of cell migration by the Drosophila PDGF/VEGF receptor. Cell 107:17-26.

20. Ishimaru S, Ueda R, Hinohara Y, Ohtani M, Hanafusa H (2004) PVR plays a critical role via JNK activation in thorax closure during Drosophila metamorphosis. EMBO J 23:3984-3994.

21. Macias A, Romero NM, Martin F, Suarez L, Rosa AL, et al. (2004) PVF1/PVR signaling and apoptosis promotes the rotation and dorsal closure of the Drosophila male terminalia. Int J Dev Biol 48:1087-1094.

22. McDonald JA, Pinheiro EM, Kadlec L, Schupbach T, Montell DJ (2006) Multiple EGFR ligands participate in guiding migrating border cells. Dev Biol 296:94-103.

23. McDonald JA, Pinheiro EM, Montell DJ (2003) PVF1, a PDGF/VEGF homolog, is sufficient to guide border cells and interacts genetically with Taiman. Development 130:3469-3478.

24. Bond D, Foley E (2009) A quantitative RNAi screen for JNK modifiers identifies Pvr as a novel regulator of Drosophila immune signaling. PLoS Pathog 5:e1000655.

25. Agaisse H, Petersen UM, Boutros M, Mathey-Prevot B, Perrimon N (2003) Signaling role of hemocytes in Drosophila JAK/STAT-dependent response to septic injury. Dev Cell 5:441-450.

26. Dostert C, Jouanguy E, Irving P, Troxler L, Galiana-Arnoux D, et al. (2005) The Jak-STAT signaling pathway is required but not sufficient for the antiviral response of drosophila. Nat Immunol 6:946-953.

27. Quintin S, Gally C, Labouesse M (2008) Epithelial morphogenesis in embryos: asymmetries, motors and brakes. Trends Genet 24:221-230.

28. Bromann PA, Korkaya H, Courtneidge SA (2004) The interplay between Src family kinases and receptor tyrosine kinases. Oncogene 23:7957-7968.

29. Singh J, Aaronson SA, Mlodzik M (2010) Drosophila Abelson kinase mediates cell invasion and proliferation through two distinct MAPK pathways. Oncogene 29:4033-4045.

30. Biteau B, Karpac J, Supoyo S, Degennaro M, Lehmann R, et al. (2010) Lifespan extension by preserving proliferative homeostasis in Drosophila. PLoS Genet 6:e1001159.
