# Supplementary material for: Novel Protein Kinase Signaling Systems Regulating Lifespan Identified by Small Molecule Library Screening Using Drosophila
Source: PLoS One. 2012 Feb 20;7(2):e29782. doi: 10.1371/journal.pone.0029782 (PMC3282711; doi:10.1371/journal.pone.0029782)
Supplement: Table S4 — Summary of food consumption of treated and untreated flies as measured by FPAs. (DOC) [file pone.0029782.s012.doc]

**Table S4.** Summary of food consumption of treated and untreated flies as measured by FPAs.

| **Drug Treatment** | **Control  (Plaques/cm^2^/fly/24 hrs)** | **Treated (Plaques/cm^2^/fly/24 hrs)** | **Significance^a^** |
| --- | --- | --- | --- |
| Tyrphostin AG 1478 (C6) | 0.057 ± 0.013 | 0.050 ± 0.012 | NS |
| Tyrphostin 1 (C4) | 0.053 ± 0.014 | 0.051 ± 0.003 | NS |
| Tyrphostin 9 (C8) | 0.053 ± 0.014 | 0.056 ± 0.004 | NS |
| PD-98059 (B1) | 0.071 ± 0.009 | 0.068 ± 0.024 | NS |
| Staurosporine (B6) | 0.063 ± 0.019 | 0.054 ± 0.027 | NS |
| SB-203580 (B3) | 0.066 ± 0.027 | 0.079 ± 0.057 | NS |
| AG-490 (D1) | 0.084 ± 0.017 | 0.087 ± 0.005 | NS |
| HA-1004 (E1) | 0.071 ± 0.009 | 0.0698 ± 0.016 | NS |
| HA-1077 (E2) | 0.071 ± 0.009 | 0.072 ± 0.014 | NS |
| KN-93 (E5) | 0.084 ± 0.017 | 0.098 ± 0.009 | NS |
| Everolimus (H8) | 0.107 ± 0.039 | 0.129 ± 0.016 | NS |
| Erbstatin analog (G1) | 0.088 ± 0.033 | 0.095 ± 0.009 | NS |
| Quercetin Dehydrate (G2) | 0.088 ± 0.033 | 0.105 ± 0.011 | NS |
| SP600125 (G8) | 0.053 ± 0.009 | 0.041 ± 0.025 | NS |
| Bay-11-7082 (G5) | 0.053 ± 0.009 | 0.054 ± 0.012 | NS |
| Indirubin (G9) | 0.084 ± 0.017 | 0.091 ± 0.009 | NS |

^a^Data were analyzed using t-tests. NS indicates the results were not significantly different than control.
