# Supplementary material for: Novel Protein Kinase Signaling Systems Regulating Lifespan Identified by Small Molecule Library Screening Using Drosophila
Source: PLoS One. 2012 Feb 20;7(2):e29782. doi: 10.1371/journal.pone.0029782 (PMC3282711; doi:10.1371/journal.pone.0029782)
Supplement: Table S5 — Summary of food consumption of treated and untreated flies as measured using CAFE assays. (DOC) [file pone.0029782.s013.doc]

**Table S5.** Summary of food consumption of treated and untreated flies as measured using CAFE assays.

| **Drug Treatment** | **Control  (μL/fly/24 hrs)** | **Treated (μL/fly/24 hrs)** | **Significance^a^** |
| --- | --- | --- | --- |
| Tyrphostin 1 (C4) | 0.49 ± 0.094 | 0.60 ± 0.054 | NS |
| HA-1004 (E1) | 0.43 ± 0.098 | 0.46 ± 0.085 | NS |
| HA-1077 (E2) | 0.43 ± 0.12 | 0.46 ± 0.11 | NS |
| Quercetin dihydrate (G2) | 0.49 ± 0.094 | 0.47 ± 0.041 | NS |

^a^Data were analyzed by one way ANOVA. NS indicates the results were not significantly different than control.
