# Supplementary material for: Novel Protein Kinase Signaling Systems Regulating Lifespan Identified by Small Molecule Library Screening Using Drosophila
Source: PLoS One. 2012 Feb 20;7(2):e29782. doi: 10.1371/journal.pone.0029782 (PMC3282711; doi:10.1371/journal.pone.0029782)
Supplement: Table S6 — Summary of the drug effects on fecal plaque size. (DOC) [file pone.0029782.s014.doc]

**Table S6.** Summary of the drug effects on fecal plaque size.

|  | **Control plaque size (mm)^a^** | **Tyrphostin 1 (C4) plaque size (mm)^b^** | **Quercetin (G2)** |
| --- | --- | --- | --- |
| **Mean ± Standard Deviation** | 0.078 ± 0.024 | 0.081 ± 0.021 | 0.079 ± 0.017 |
| **Significance** |  | NS^c^ | NS |

^a^Fly bottles used for the FPAs were positioned under a Celestron Handheld Digital Microscope, Model #44302-A, to visualize the plaques. The diameter of the plaques was determined using the software provided with the microscope (n=44 for each condition).

^b^Tyrphostin 1 and quercetin were present in the medium at 0.1 and 1.0 mM, respectively.

^c^NS indicates the treatment results were not significantly different than control as determined by one way ANOVA.
