# Supplementary material for: Novel Protein Kinase Signaling Systems Regulating Lifespan Identified by Small Molecule Library Screening Using Drosophila
Source: PLoS One. 2012 Feb 20;7(2):e29782. doi: 10.1371/journal.pone.0029782 (PMC3282711; doi:10.1371/journal.pone.0029782)
Supplement: Supporting Information S1 — Supporting Materials and Methods. (DOC) [file pone.0029782.s015.doc]

# Supporting Information S1

**Supporting Materials and Methods**

**Large scale production of *Drosophila* for studies.** Wild-type Oregon-R-C *Drosophila* were maintained on SCM at 25 °C with a 12 h light/dark cycle and 60% relative humidity. SCM was prepared by boiling 126 g of inactive dry yeast (Genesee Scientific), 28 g agar (Genesee Scientific), 72 g glucose (VWR), 218 g sucrose (Costco), and 2400 ml of RO water for 15 minutes in an open pan with constant stirring. A solution of 180 g cornmeal (Quaker corn meal, Genesee Scientific), 1.6 g CaCl_2_, in 400 ml RO water was incubated at room temperature for 15 minutes, mixed, and added to the boiling solution, followed by additional 15 minutes of boiling with constant stirring. The solution was allowed to cool to 60°C, and 15.9 ml of propionic acid, 1.6 ml of phosphoric acid (85%), and 7.2 g of Tegosept (Genesee Scientific), dissolved in 50 ml of 70% ethanol, were added to the solution with stirring. Approximately 75 ml of this liquid medium was poured into 8 ounce fly bottles (Genesee Scientific). The bottles were covered with layers of cheese cloth, left at room temperature overnight, and closed with a cotton flug (Genesee Scientific). Thirty to 50 *Drosophila* of both sexes were incubated in these bottles at room temperature for approximately 24 hours to allow mating and egg laying. After removal of these flies, the bottles were incubated at room temperature for an additional 9 to 10 days. Freshly eclosed flies of both sexes were collected over a 24-h period.

For the collection of larger numbers of embryos, approximately 50 mls of adult *Drosophila* (approximately 7500 flies) were collected from 8 ounce fly bottles prepared as described above, and released into a Plexiglas population cage (8.5 x 8.5 x 26.7 cm) housing an open tray (30 cm x 19 cm x 1 cm) containing 300 ml of Apple Juice-Agar Medium covered by a thin layer of active dry yeast paste (Red Star) suspended with water in a thick slurry. The tray of Apple Juice-Agar Medium was prepared by boiling 5 L of water containing 200 g agar, reducing the heat to low, adding 1.6 L apple juice, 165 g dextrose, and 9 g Tegosept which had been dissolved in 50 ml 70% ethanol. The mixture was stirred for 15 minutes, and 300 mls poured into the trays. The medium was allowed to cool to room temperature. The trays were enclosed in a plastic bag and stored at 4°C until used. Before use, the Apple Juice Agar Medium in the trays was spread with active dry yeast paste and placed in the population cages. Female flies lay eggs on the food in the trays while eating. The food trays were removed and replaced with new trays every 10-12 h for collection of embryos. Embryos were collected from the trays by flushing the contents, using RO water and a soft brush, through a 1 mm stainless steel analysis sieve [Prüfsieb #502801] to remove debris. *Drosophila* embryos were then collected by passage of the solution through a 100 µm stainless steel analysis sieve [Retsch, Haan, Germany, #5581856]. The collected embryos were washed in the sieve with RO water, and air dried at room temperature before weighing.

Two methods were used to hatch flies from the embryos. For the first method, “embryo boxes” constructed according to the unpublished method of Dennis Spann (personal communication). Rubbermaid 17 cup Serving Saver boxes (30 x 21 x 8 cm) were modified with a fine mesh, nylon screen (18 x 8 cm) attached securely to the lid for ventilation. A double layer of cotton was spread in the bottom of the boxes, and yeast food mixture (108g sucrose, 3.0 ml 85% phosphoric acid, 0.525 ml propionic acid, 1.4 g Tegosept in 12 ml 70% ethanol, and 216 g of active dry yeast) was added to 570 ml Nanopure ultrafiltered RO water (Nano-pure water; Barnstead, Inc.) and poured onto the cotton. The box was covered with the lid, and fermented at room temperature for 5 to 6 hours. During the fermentation the cotton rose due to the CO_2_ produced, and was pushed down several times. The dried *Drosophila* embryos (0.25 g) were placed onto 5, 70 mm circles of Whatman #1 filter paper, wetted with a spray bottle of RO water, and gently spread over the filter with a soft paint brush. These filters were placed on top of the cotton/yeast layer, pierced multiple times with a dissecting needle, and incubated for 10.5 days with a 12 hour light/dark cycle, at 60% humidity and 25˚C. When flies begin to emerge, they were lightly anesthetized with CO_2_, and collected onto a tray. Approximately 50 flies were distributed into 8 ounce fly bottles containing SCM.

Another method of incubating *Drosophila* embryos was also used [1]. Briefly, the embryos collected from the Plexiglas population cage were transferred into a 15 ml centrifuge tube (Falcon) and washed by resuspension and settling 3 times in ~10 ml of PBS. After removal of the final supernatant, 13 µl aliquots of the embryo pellet were transferred using a cutoff pipetman tip to an 8 ounce fly bottle containing SCM, prepared as described above. The fly bottles described above were incubated at 25°C for 9 to 10 days until flies emerged. The males were separated from female flies under light CO_2_ anesthesia and 50 male flies were placed in 8 ounce fly bottles. With this method the newly emerged flies do not need to be anesthetized with CO_2_ and more flies emerge. This method was preferred for its relative simplicity and reproducibility.

***Drosophila* lifespan studies.** Lids of 35 x 10 mm Petri dishes containing ~5.5 ml of SCM were stored covered at 4°C until used. The SCM containing lids were warmed to room temperature, and 10 µl DMSO containing the indicated concentrations of drugs were applied to the surface of the SCM and spread carefully over the entire SCM surface. Controls utilized 10 µl of DMSO spread as described above. Studies using dyes in the DMSO indicate that only minimal diffusion (approximately 1 mm) into the SCM occurs. A total of 4 bottles per drug concentration, with 50 male flies per bottle (200 flies total) were used for each determination. The lids were attached to the bottle opening and secured with tape. The walls of the plastic bottles were pierced with small air holes. The bottles were incubated with the lid closures on top (to minimize evaporative drying) at 25 °C, 60% humidity, and with a 12:12 hour light:dark cycle. The SCM-lids were replaced twice weekly. The number of flies surviving at each time point was determined. CO_2_ anesthesia was not used after the initial sorting of males from females.

# References

1. Clancy DJ, Kennington WJ (2001) A simple method to achieve consistent larval density in bottle cultures. Dros Inf Serv 84:168.
